# Renal outcomes according to renal replacement therapy modality and treatment protocol in the ATN and RENAL trials

**DOI:** 10.1186/s13054-022-04151-5

**Published:** 2022-09-06

**Authors:** Thummaporn Naorungroj, Ary Serpa Neto, Amanda Wang, Martin Gallagher, Rinaldo Bellomo

**Affiliations:** 1 Department of Intensive Care Medicine, Austin Hospital, 145 Studley Road, Heidelberg, Melbourne, VIC 3084 Australia; 2 Department of Intensive Care, Faculty of Medicine Siriraj Hospital, Mahidol University, Bangkok, Thailand; 3 Australian and New Zealand Intensive Care Research Centre Monash University, Melbourne, Australia; 4 Department of Critical Care Medicine, Hospital Israelita Albert Einstein, São Paulo, Brazil; 5 Department of Critical Care, The University of Melbourne, Melbourne, Australia; 6 Data Analytics Research and Evaluation (DARE) Centre, Austin Hospital, Melbourne, Australia; 7 Department of Intensive Care, Royal Melbourne Hospital, Melbourne, Australia; 8 The George Institute for Global Health, University of New South Wales, Sydney, Australia; 9 Concord Clinical School, The University of Sydney, Sydney, Australia; 10 Department of Renal Medicine, Concord Repatriation General Hospital, Concord West, Australia; 11 Department of Nephrology, Liverpool Hospital, Sydney, Australia

**Keywords:** Acute kidney injury, Continuous renal replacement therapy, Intermittent hemodialysis, Mortality, Dialysis dependence

## Abstract

**Background:**

In critically ill patients with acute kidney injury, renal replacement therapy (RRT) modality and treatment protocols may affect kidney recovery. This study explored whether RRT modality and treatment protocol affected RRT dependence in the ‘Randomized Evaluation of Normal versus Augmented Level of RRT’ and the ‘Acute Renal Failure Trial Network’ (ATN) trials.

**Methods:**

Primary outcome was 28-day RRT dependence. Secondary outcomes included RRT dependence among survivors and in different SOFA-based treatment protocol groups. We used the Fine-Gray competing-risk model sub-distribution hazard ratio (SHR) to assess the primary outcome. Analyses were adjusted for confounders.

**Results:**

Of 2542 patients, 2175 (85.5%) received continuous RRT (CRRT) and 367 (14.4%) received intermittent hemodialysis (IHD) as first RRT modality. CRRT-first patients had greater illness severity. After adjustment, there was no between-group difference in 28-day RRT dependence (SHR, 0.96 [95% CI 0.84–1.10]; *p* = 0.570) or hospital mortality (odds ratio [OR], 1.14 [95% CI 0.86–1.52]; *p* = 0.361) However, among survivors, CRRT-first was associated with decreased 28-day RRT dependence (OR, 0.54 [95% CI 0.37–0.80]; *p* = 0.002) and more RRT-free days (common OR: 1.38 [95% CI 1.11–1.71]). Moreover, among CRRT-first patient, the ATN treatment protocol was associated with fewer RRT-free days, greater mortality, and a fourfold increase in RRT dependence at day 28.

**Conclusions:**

There was no difference in RRT dependence at day 28 between IHD and CRRT. However, among survivors and after adjustment, both IHD-first and the ATN treatment protocol were strongly associated with greater risk of RRT dependence at 28 days after randomization.

*Trial registration* NCT00221013 registered September 22, 2005, and NCT00076219 registered January 19, 2004.

**Supplementary Information:**

The online version contains supplementary material available at 10.1186/s13054-022-04151-5.

## Introduction

Acute kidney injury (AKI) treated with renal replacement therapy (RRT) carries a high mortality [1]. Among survivors, renal recovery is a key outcome, because of the adverse impact of continued RRT on later morbidity and mortality [2, 3].

A current set of guidelines recommends either continuous RRT (CRRT) or intermittent hemodialysis (IHD) as complementary therapies for severe AKI [4]. However, RRT modality may affect subsequent RRT dependence and RRT-free days. Regrettably, only a few underpowered randomized clinical trials (RCT) have investigated the impact of RRT modality on renal recovery, leaving the issue unresolved [5, 6]. A recent meta-analysis found that CRRT was associated with decreased RRT dependence among survivors compared to IHD [7] but lacked the ability to adjust for individual illness severity, diagnosis, baseline physiological state, and premorbid characteristics. The availability of such detailed individual data is necessary to make more meaningful comparisons. In addition to RRT modality, treatment protocols may also influence renal and patient outcomes, but their outcome association has never been studied.

Accordingly, we aimed to compare the rate of persistent RRT dependence (defined as RRT dependence at 28 days after randomization) in patients first treated with CRRT vs patients first treated with IHD among subjects enrolled in the ‘Randomized Evaluation of Normal vs. Augmented Level of RRT’ (RENAL) and the ‘Acute Renal Failure Trial Network’ (ATN) trials [8, 9]. Moreover, we aimed to study the association of treatment protocols with renal and patient outcomes. We hypothesized that RRT modality and treatment protocol would affect recovery to RRT independence.

## Methods

### Study design

We conducted a pooled analysis of individual patient data from the RENAL [8] and ATN [9] trials. The RENAL trial was a multicenter RCT comparing two different intensities of CRRT (40 mL/kg/h vs. 25 mL/kg/h) for AKI in 35 intensive care units (ICU) in Australia and New Zealand (ANZ) [8]. The ATN trial was a multicenter RCT comparing the efficacy of two different intensities of RRT (35 mL/kg/h vs. 20 mL/kg/h) for AKI in 27 ICUs in the USA [9].

### Patients

All patients included in the original trials were considered for inclusion in the present study. Briefly, patients were eligible to participate in the RENAL trial if they were critically ill adults with AKI, were deemed to require CRRT by the attending physician, and fulfilled at least one of predefined inclusion criteria (severe organ edema, oliguria, hyperkalemia, uremia, and/or severe metabolic acidosis). In the ATN trial, critically ill adults with AKI, deemed to require RRT, and with at least one non-renal organ failure or sepsis were included. The only exclusion criterion in the present analysis was the absence of sufficient information on the first modality of RRT after randomization.

### Data collection and definitions

We established the exposure, CRRT or IHD, according to the first post-randomization RRT modality as a quasi-equivalent to intention to treat analysis. Illness severity was assessed by Acute Physiology and Chronic Health Evaluation (APACHE) III either directly recorded or derived from APACHE II using predefined formulas [10].

### Outcomes

The primary outcome was RRT dependence at day 28 after randomization, defined as the last date of RRT. Secondary outcomes included RRT dependence at day 60, RRT dependence at day 28 and day 60 among survivors, RRT-free days at day 28 (patient alive and free of RRT), ICU, and hospital length of stay, and ICU, hospital, 28- and 60-day mortality.

### Statistical analysis

Categorical variables are presented as numbers and percentages and compared using Fisher exact tests. Continuous variables are presented as median (quartile 25%–quartile 75%) and compared using Wilcoxon rank-sum test. Missingness is presented in Additional file 1: Table S1.

All analyses were adjusted using baseline data (Additional file 1: Table S2) to develop a model for the risk of being RRT dependent at 28 days.

The primary outcome was first compared between the groups with a Fine-Gray competing-risk model, with death before the RRT independence as competing risk. Results are shown as sub-distribution hazard ratios (SHR) with their 95% confidence interval (CI) and presented as cumulative incidence plots. The same strategy was used for RRT dependence at day 60, ICU, and hospital length of stay. In the competing-risk models, subjects who died remain in the risk set and the RRT-free days at day 28 were compared using cumulative logistic models and presented as a common odds ratio (OR) with 95% CI. In this model, a common OR > 1.00 indicates a greater chance of having more RRT-free days at day 28 (better outcome). ICU and hospital mortality were compared with generalized linear models with binomial distribution and presented as odds ratios (ORs) and 95% CI. 28- and 60-day mortality were compared with Cox proportional hazard model and presented as hazard ratios (HRs) and 95% CI. The proportional hazard assumption was assessed through Schoenfeld residuals.

In a sensitivity analysis, to assess the consistency of findings, a covariate-balancing propensity score (CBPS) was used to control for observed confounding factors that may influence outcomes [11]. The CBPS was estimated for each patient with logistic regression using the variables described above. Using this CBPS, the following additional analyses were performed: (1) weighted regression by the inverse probability of treatment weighting; and (2) weighted regression by stabilized inverse probability of treatment weighting [12] (Additional file 1: Methods).

To confirm or refute the findings in different cohorts, the primary and secondary outcomes were re-assessed in the following groups: (1) patients receiving CRRT or IHD exclusively in the first three days; (2) patients receiving CRRT or IHD exclusively during the whole follow-up; and (3) patients with a cardiovascular SOFA score of ≤ 2 at randomization.

In addition to study the possible association with trial protocol with renal and patient outcomes in patients who received CRRT first by protocol both in ATN and RENAL, we assessed those patients with cardiovascular SOFA score of ≥ 3 at randomization. In such patients, after CRRT-first start, the RENAL trial protocol prescribed continuing CRRT until day 28 or ICU discharge, while the ATN trial prescribed transition to IHD when vasopressor support was no longer needed.

In a final sensitivity analysis, for key secondary outcomes, RRT dependence at day 28 and 60 were also assessed considering only patients alive at latest follow-up. In this group of patients, the comparison of RRT dependence at day 28 and 60 among survivors was done considering a generalized linear model with binomial distribution. We also analyzed the relationship between IHD/CRRT time ratio and RRT dependence at day 28. An IHD/CRRT time ratio defined as the percentage of overall RRT time that the patients spent on IHD and CRRT until day 28 after randomization.

For all the models, variables with less than 3% of missing were imputed by the median value of the overall cohort. Missing data in premorbid estimated glomerular filtration rate were imputed based on creatinine levels according to age and sex [13]. All analyses were conducted in R v.4.0.2 (R Foundation, Vienna, Austria). Given the multiplicity of comparisons, to attenuate the risk of type I error and yet maintain sufficient sensitivity as part an exploratory analysis, we chose to set the significance level at < 0.01.

## Results

### Patients

The study trials enrolled a total of 2589 patients. After the exclusion of 16 patients with missing data for first RRT modality, and 31 patients with missing primary outcome, we included 2542 patients with 2175 (85.5%) in the CRRT-first group and 367 (16.5%) in the IHD-first group (Additional file 1: Fig. S1).

Baseline characteristics are shown in Table 1. CRRT-first patients were older, more often female, had higher APACHE III and cardiovascular SOFA scores, received their RRT earlier after ICU admission, had more acidemia and hyperkalemia, less sepsis and received more mechanical ventilation.

**Table 1:** Baseline characteristics of the included cohort

	CRRT (*n* = 2175)	IHD (*n* = 367)	*p* value
Age, years	65.0 (54.0–75.0)	61.0 (50.5–71.0)	< 0.001
Male gender—no. (%)	1429 (65.7)	279 (76.0)	< 0.001
Weight, kilograms	80.0 (70.0–90.4)	83.0 (71.9–96.5)	0.009
Type of admission—no. (%)			< 0.001
Medical	1351 (62.1)	202 (55.0)	
Surgical	729 (33.5)	132 (36.0)	
Other	95 (4.4)	33 (9.0)	
APACHE III	97.0 (80.0–113.5)	76.6 (65.8–95.1)	< 0.001
Cardiovascular SOFA	4.0 (2.0–4.0)	0.0 (0.0–2.0)	< 0.001
0	298 (13.7)	212 (57.8)	< 0.001
1	234 (10.8)	61 (16.6)	
2	67 (3.1)	28 (7.6)	
3	425 (19.6)	17 (4.6)	
4	1148 (52.9)	49 (13.4)	
Hours between randomization and start of treatment	31.0 (10.0–79.0)	72.0 (24.0–144.0)	< 0.001
Premorbid creatinine, µmol/L	99.0 (79.6–132.6)	97.2 (70.7–132.6)	0.047
Estimated glomerular filtration rate, mL/min	61.2 (42.6–84.1)	67.5 (50.1–96.8)	< 0.001
Premorbid creatinine imputed, µmol/L*	100.7 (84.3–114.9)	100.7 (79.6–114.9)	0.338
Estimated glomerular filtration rate imputed, mL/min*	49.6 (41.8–60.4)	49.7 (40.6–65.1)	0.122
Diabetes—no. (%)	186 (26.0)	124 (33.8)	0.009
At baseline—no. (%)			
Mechanical ventilation	1713 (78.8)	227 (61.9)	< 0.001
Sepsis	1173 (54.0)	222 (60.5)	0.023
Oliguria	1459 (67.1)	257 (70.0)	0.292
Hyperkalemia	123 (5.7)	4 (1.1)	< 0.001
Acidemia	572 (27.3)	15 (4.6)	< 0.001
Urea > 25 mmol/L	843 (38.8)	115 (31.5)	0.010
Creatinine > 300 µmol/L	1087 (50.0)	208 (57.0)	0.015
High intensity group—no. (%)	1084 (49.8)	180 (49.0)	0.822
Laboratory tests at baseline			
Urea, mmol/L	20.3 (13.7–30.6)	19.3 (13.6–27.5)	0.031
Creatinine, µmol/L	300.0 (212.2–410.0)	309.4 (238.7–415.5)	0.121
pH	7.30 (7.21–7.37)	7.36 (7.29–7.42)	< 0.001
Bicarbonate, mmol/L	19.0 (16.0–23.0)	22.0 (18.4–25.0)	< 0.001

Data are median (quartile 25–quartile 75%) or No (%). Percentages may not total 100 because of rounding. Denominators are shown when the overall sample size was not available

*CRRT* Continuous renal replacement therapy; *IHD* Intermittent hemodialysis; *APACHE* Acute physiology and chronic health evaluation; *SOFA* Sequential organ failure assessment; *ICU* intensive care unit

*Missing values in creatinine imputed according to age and gender (Tiao JYH, et al. Cardiovascular Surgery 2002;10:445–51)

### Process of care

CRRT patients were followed up in ICU for a median of 9 days compared with 12 days) for IHD-first patients (Additional file 1: Table S3). CRRT-first patients received CRRT for a median of 5 days. IHD-first patients received the IHD for a median of 6 days. Overall, 77.4% CRRT-first patients received CRRT exclusively during the available follow-up versus 76.6% IHD-first patients. The percentage of patients receiving CRRT or IHD in each group over time is shown in Additional file 1: Figs. S2 and S3.

### Primary outcome

**Fig. 1:**
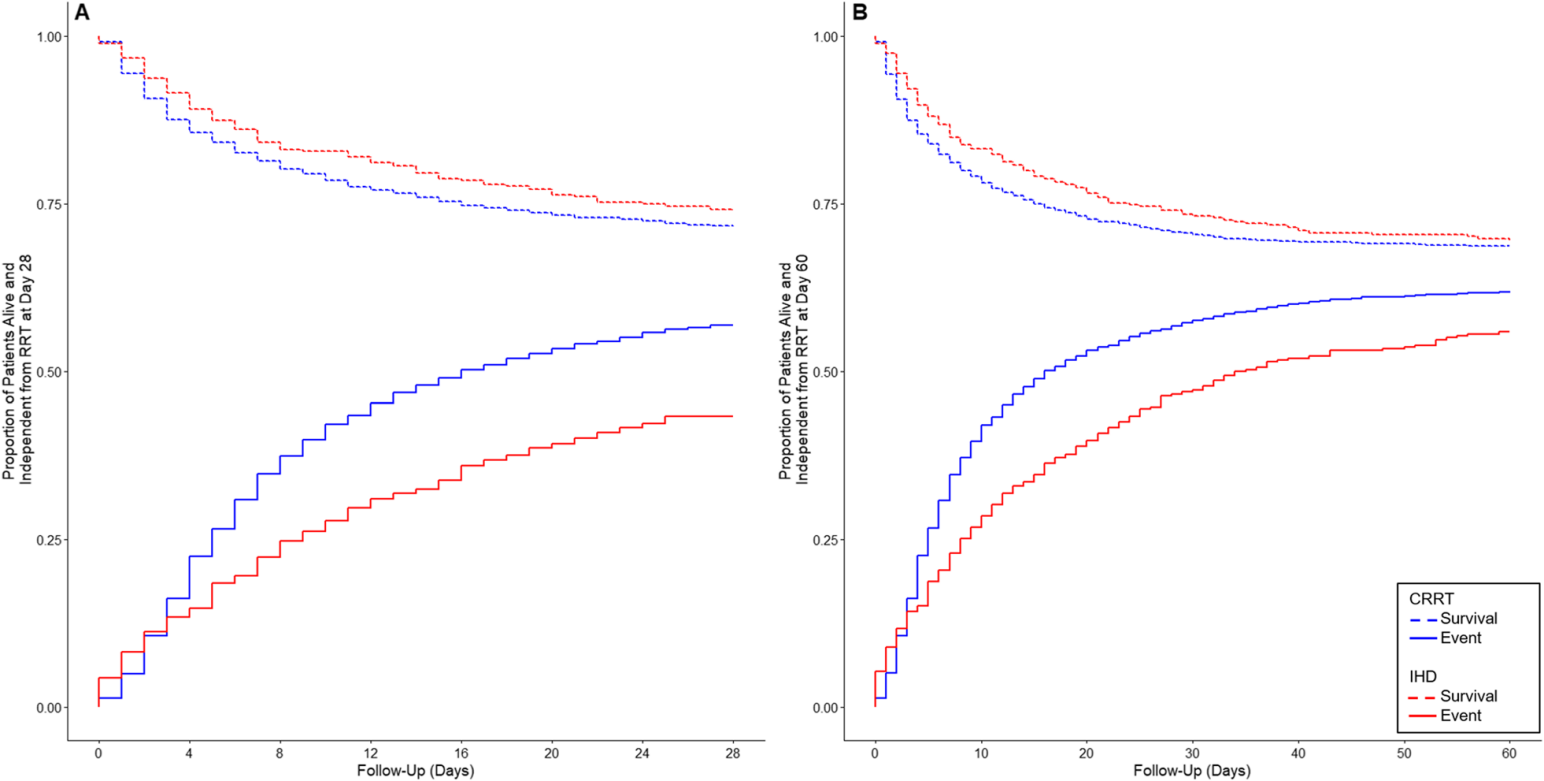
Cumulative Incidence Plot of Renal Replacement Therapy Independence at Day 28 and 60 before and after Matching. Panel A, renal replacement therapy independence at day 28 in the cohort before the covariate-balancing propensity score matching. Panel B, renal replacement therapy independence at day 60 in the cohort before the covariate-balancing propensity score matching. Adjusted models in panel A and B included for age, gender, weight, type of admission (medical, surgical or other), APACHE III, cardiovascular SOFA, hours between randomization and therapy, use of mechanical ventilation, presence of oliguria, presence of hyperkalemia, presence of sepsis, last bicarbonate, urea and creatinine before randomization, premorbid estimated glomerular filtration rate, and intensity of treatment (as allocated in the original trials). SHR denotes sub-distribution hazard ratio and CI confidence interval

**Fig. 2:**
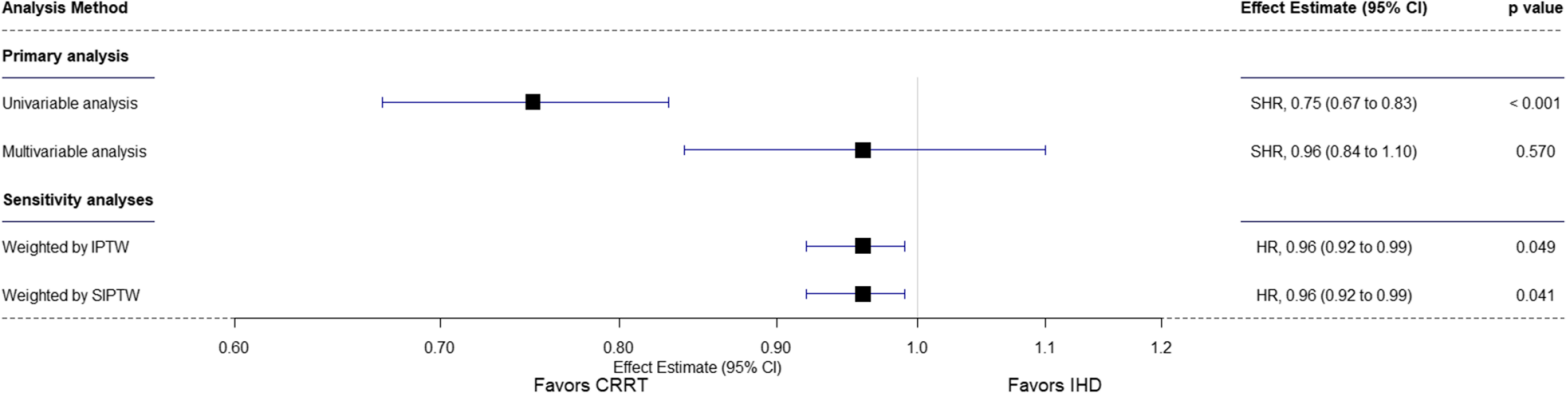
Forest Plot for the Analyses of the Primary Outcome. Multivariable analysis in the primary analysis is adjusted for age, gender, weight, type of admission (medical, surgical or other), APACHE III, cardiovascular SOFA, hours between randomization and therapy, use of mechanical ventilation, presence of oliguria, presence of hyperkalemia, presence of sepsis, last bicarbonate, urea and creatinine before randomization, premorbid estimated glomerular filtration rate, and intensity of treatment (as allocated in the original trials). IPTW inverse probability of treatment weighting, SIPTW stabilized inverse probability of treatment weighting, CI confidence interval, SHR sub-distribution hazard ratio, and HR hazard ratio

On unadjusted analysis, at day 28, 320 (14.7%) CRRT-first patients and 113 (30.8%) IHD-first group were alive and RRT dependent (SHR, 0.75 [95% CI 0.67–0.83]; *p* < 0.001) (Table 2, Fig. 1 and Fig. 2). After adjustment, however, this difference was not statistically significant (SHR, 0.96 [95% CI 0.84–1.10]; *p* = 0.570).

**Table 2:** Primary and secondary outcomes in the included cohort

	CRRT (*n* = 2175)	IHD (*n* = 367)	Unadjusted analysis		Adjusted analysis^a^	
			Effect estimate (95% CI)	*p* value	Effect estimate (95% CI)	*p* value
*Primary outcome*						
RRT dependence at day 28—no. (%)	320 (14.7)	113 (30.8)	SHR, 0.75 (0.67 to 0.83)	< 0.001	SHR, 0.96 (0.84 to 1.10)	0.570
Death before the event	618 (28.4)	95 (25.9)				
*Secondary outcomes*						
RRT dependence at day 60—no. (%)	145 (6.8)	49 (13.7)	SHR, 0.75 (0.62 to 0.90)	0.002	SHR, 1.04 (0.85 to 1.28)	0.670
Death before the event	673 (31.3)	109 (30.4)				
RRT-free days at day 28	0.0 (0.0–22.0)	0.0 (0.0–17.0)	COR, 1.38 (1.11 to 1.71)	0.004	COR, 1.45 (1.12 to 1.88)	0.005
Mean ± SD	9.1 ± 10.9	7.2 ± 10.2				
ICU length of stay, days	9.0 (4.0–19.0)	13.0 (7.0–25.0)	SHR, 0.82 (0.72 to 0.94)*	0.003	SHR, 1.03 (0.87 to 1.21)*	0.750
In survivors	11.0 (6.0–21.0)	13.0 (7.0–25.0)				
Hospital length of stay, days	19.0 (9.0–36.0)	25.0 (15.0–41.0)	SHR, 0.69 (0.60 to 0.81)*	< 0.001	SHR, 0.81 (0.68 to 0.97)*	0.025
In survivors	28.0 (17.0–47.0)	30.0 (18.0–44.0)				
ICU mortality—no. (%)	848 (39.0)	108 (29.4)	OR, 1.53 (1.21 to 1.95)	< 0.001	OR, 0.96 (0.71 to 1.29)	0.778
Hospital mortality—no. (%)	1016 (46.7)	121 (33.0)	OR, 1.78 (1.41 to 2.26)	< 0.001	OR, 1.14 (0.86 to 1.52)	0.361
28-day mortality—no. (%)	875 (40.2)	114 (31.1)	HR, 1.38 (1.13 to 1.68)**	0.001	HR, 0.90 (0.72 to 1.12)**	0.343
60-day mortality—no. (%)	1005 (46.7)	143 (39.4)	HR, 1.28 (1.07 to 1.52)**	0.006	HR, 0.92 (0.75 to 1.13)**	0.434
*Sensitivity analysis*						
Among survivors at the longest follow-up—no. (%)						
RRT dependence at day 28	232 (20.9)	90 (40.9)	OR, 0.38 (0.28 to 0.52)	< 0.001	OR, 0.54 (0.37 to 0.80)	0.002
RRT dependence at day 60	136 (12.3)	49 (22.6)	OR, 0.48 (0.33 to 0.70)	< 0.001	OR, 0.76 (0.48 to 1.22)	0.261

Data are median (quartile 25–quartile 75%) or No (%). Percentages may not total 100 because of rounding. Denominators are shown when the overall sample size was not available

*CRRT* Continuous renal replacement therapy; *IHD* Intermittent hemodialysis; *ICU* Intensive care unit; *RRT* Renal replacement therapy; *SHR* Sub-distribution hazard ratio; *MD* Mean difference; *RRT* Renal replacement therapy; *HR* Hazard ratio; *OR* Odds ratio; *COR* Common odds ratio

^a^All models adjusted for age, gender, weight, type of admission (medical, surgical, or other), APACHE III, cardiovascular SOFA, hours between randomization and therapy, use of mechanical ventilation, presence of oliguria, presence of hyperkalemia, presence of sepsis, last bicarbonate, urea and creatinine before randomization, premorbid estimated glomerular filtration rate, and intensity of treatment (as allocated in the original trials)

^*^ICU and hospital length of stay censored at day 60

^**^p value for Schoenfeld residual is 0.830 for 28-day mortality and 0.260 for 60-day mortality

### Secondary outcomes

**Fig. 3:**
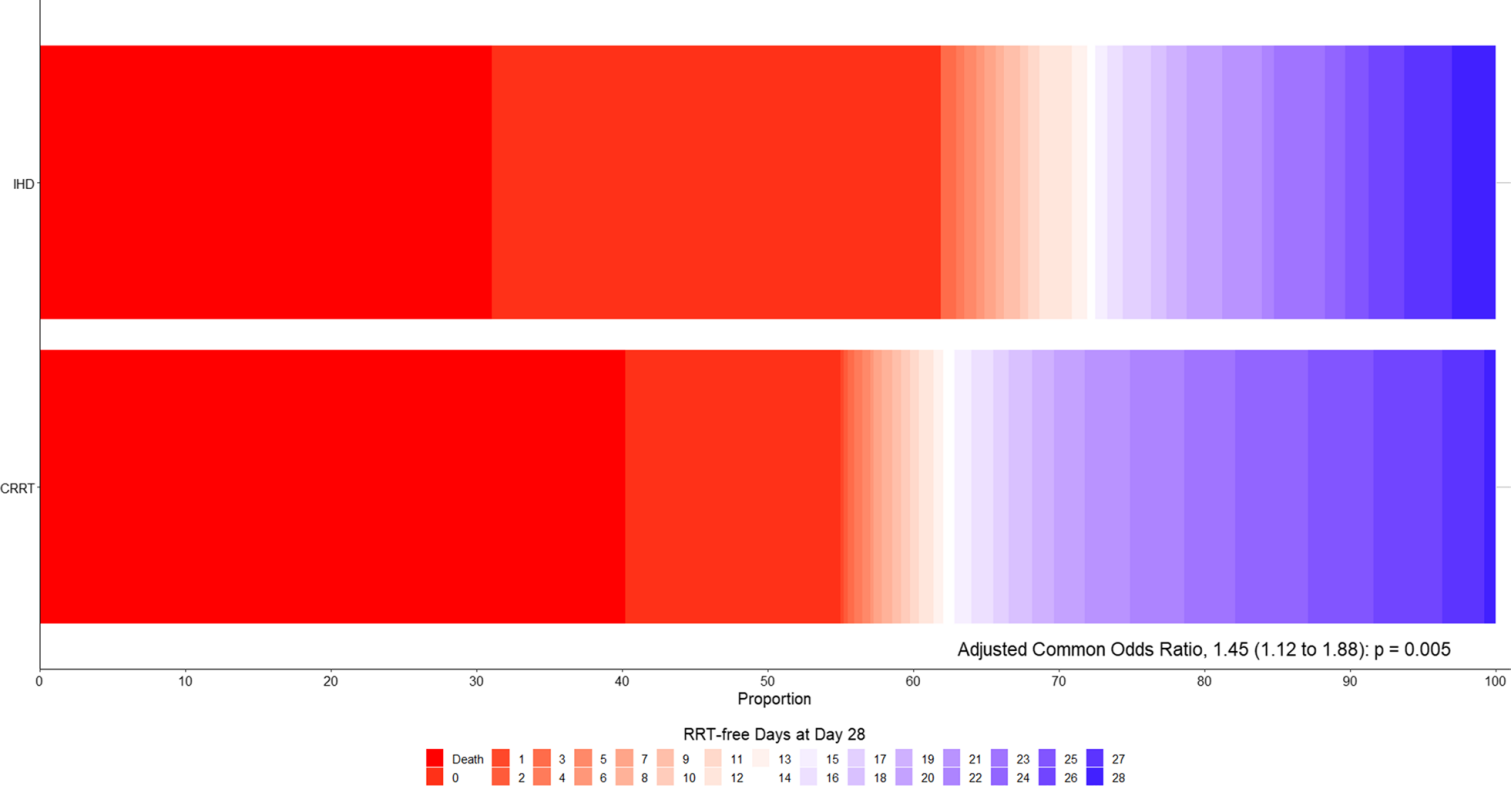
Renal Replacement Therapy-Free Days at Day 28. Renal replacement therapy-free days at day 28 as horizontally stacked proportions according to study group. Red represents worse outcomes, and blue represents better outcomes. *COR is common odds ratio*

Before adjustment, CRRT-first patients had a lower incidence of RRT dependence at day 60, a longer ICU and hospital length of stay, and a higher ICU, hospital, 28-day and 60-day mortality (Table 2 and Fig. 1). After adjustment, only the number of RRT-free days at day 28 remained significantly greater among CRRT-first patients (COR, 1.45 [95% CI 1.12–1.88]; *p* = 0.005) (Table 2 and Fig. 3).

### Sensitivity analyses

Results of the sensitivity analyses for the primary outcome are shown in Fig. 2 and Additional file 1: Table S4.

Baseline characteristics of patients receiving CRRT or IHD exclusively in the first three days of follow-up or exclusively during the whole follow-up available for each patient are shown in Additional file 1: Table S5. The modalities used over this period are shown in Additional file 1: Figs. S4 and S5. Primary and secondary outcomes in both cohorts are shown in Additional file 1: Tables S6 and S7.

After adjustment, RRT dependence at day 28 was not different in patients receiving CRRT or IHD exclusively in the first three days. However, among survivors, CRRT carried a lower risk of RRT dependence at day 28 (OR, 0.42 [95% CI 0.26–0.70]; *p* < 0.001) (Additional file 1: Table S6).

**Fig. 4:**
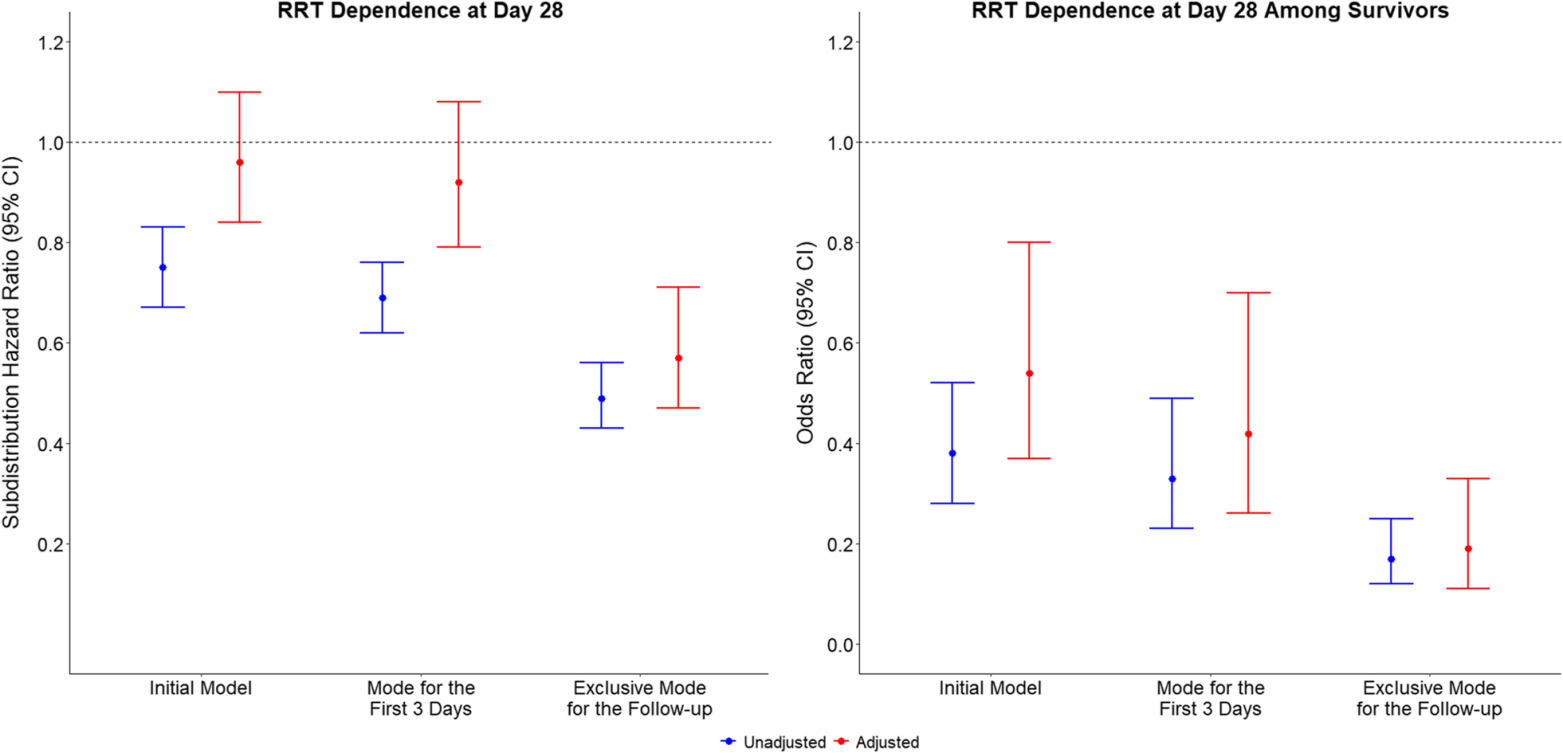
Effect of Continuous Renal Replacement Therapy on Renal Replacement Therapy Dependence at Day 28 Compared to Intermittent Hemodialysis According to the Different Cohorts Assessed. Panel A, RRT dependence at day 28 considering death as a competing risk. Panel B, RRT dependence at day 28 among survivors at the longest follow-up only. Initial mode considered the mode used after randomization. Mode for the first three days considered patients receiving CRRT or IHD exclusively in the first three days of follow-up (patients who died or do not have information of modality in the first three days were excluded from this analysis). Exclusive mode for the follow-up considered patients receiving CRRT or IHD exclusively during the whole follow-up available for each patient (including those who died early). Adjusted models included for age, gender, weight, type of admission (medical, surgical or other), APACHE III, cardiovascular SOFA, hours between randomization and therapy, use of mechanical ventilation, presence of oliguria, presence of hyperkalemia, presence of sepsis, last bicarbonate, urea and creatinine before randomization, premorbid estimated glomerular filtration rate, and intensity of treatment (as allocated in the original trials)

After adjustment, the number of RRT-free days at day 28 was greater in patients in the CRRT group. The evolution of the effect estimate according to the sensitivity cohorts is shown in Fig. 4. These analyses also found shorter duration of hospital stay when CRRT was the only modality for the first 3 days (Additional file 1: Table S6).

When only patients treated exclusively with CRRT or IHD were compared, CRRT was associated with significantly lower risk of persistent RRT dependence, a higher number of RRT-free days at day 28, and shorter ICU and hospital stay, but also greater ICU and hospital mortality. However, 28-day and 60-day mortality were not statistically different. In sensitivity analysis, among survivors, CRRT had a lower risk of RRT dependence at day 28 (OR, 0.19 [95% CI 0.11–0.33]; *p* < 0.001) (Additional file 1: Table S7).

### Sensitivity analysis among survivors

Baseline characteristics of survivors according to RRT modality are shown in Additional file 1: Table S8. The differences between the CRRT and IHD groups were similar to those of the overall cohort. However, compared with IHD-first survivors, CRRT-first survivors had an odds ratio of 0.54 (0.37–0.80) (*p* = 0.002) of remaining RRT dependent at day 28 (Table 2).

When comparing patients with a cardiovascular SOFA score of ≤ 2 (Additional file 1: Table S9), there were multiple baseline differences. After adjustment for such differences, RRT-free days at day 28 and RRT independence at day 28 among survivors were greater in CRRT-first patients (Additional file 1: Table S10).

The relationship between IHD/CRRT time ratio and RRT dependence at day 28 is shown in Additional file 1: Fig. S6. We found that after adjustment for key baseline characteristics, among survivors at day 28, patients who received a higher IHD/CRRT time ratio had an increased risk of RRT dependence at day 28.

### Assessment of treatment protocol effect

We assessed patients with a cardiovascular SOFA score of ≥ 3 separately because they received CRRT as first treatment in both trials. Among these patients, there were multiple baseline differences according to each trial (Additional file 1: Table S11). Among such CRRT-first patients in both trials, subsequent dialytic care differed by protocol (Additional file 1: Table S12). ATN patients received CRRT for longer, were more likely to subsequently receive IHD, had more days of IHD in those so treated, much greater use of IHD, and more days of treatment survivors (Additional file 1: Figs. S7 and S8)**.**

After adjustment, treatment of such cardiovascular SOFA score of ≥ 3 CRRT-first patients according to the ATN protocol was associated with greater length of stay in ICU, fewer RRT free days, longer hospital stays, significantly greater mortality, and a greater than fourfold increase in RRT dependence at day 28 and day 60 among survivors **(**Additional file 1: Table S13**)**.

## Discussion

### Key findings

This individual patient meta-analysis of the ATN and RENAL trials found that there was no statistically significant RRT dependence at day 28 among IHD- and CRRT-first modality. However, this study found multiple indications that renal recovery was affected by RRT modality: (a) overall, after adjustment, the number of RRT-free days at day 28 was significantly greater in CRRT-first patients; (b) in patients receiving CRRT exclusively in the first three days, there was a lower risk of RRT dependence at day 28 among survivors; (c) among those exclusively treated with CRRT or IHD throughout their ICU stay, there was a significantly lower risk of 28-day RRT dependence, and a higher number of RRT-free days to day 28 in CRRT patients; (d) among patients with a cardiovascular SOFA score of ≤ 2, RRT-free days at day 28, and among survivors, RRT independence at day 28 were greater in CRRT-first patients, (e) among survivors who received CRRT as their first modality, there was a significant decrease in RRT dependence at day 28 and (f) among survivors, a higher IHD/CRRT time ratio was associated with RRT dependence at day 28. Finally, irrespective of first choice of RRT modality, as seen in those patients with a cardiovascular score ≥ 3 who all received CRRT-first therapy in both trials, after adjustment, the different trial protocols were associated with markedly different renal and patient outcomes.

### Relationship with previous studies

Many aspects of RRT in ICU patients remain controversial including the effect of modality on renal recovery [14–16]. Yet, it is clinically plausible that RRT modality might affect this outcome [7, 17]. Unfortunately, all previous analyses could not adjust for individual baseline characteristics. The databases of the ATN and RENAL trial, however, enable such adjustments. In this regard, the ATN database has been recently used to compare daily with second daily IHD (treatments assigned by randomization) and found that second daily IHD was associated with more RRT-free days and renal recovery at 28 days, implying that greater IHD exposure delays renal recovery [18]. Our findings are aligned with such observations.

When comparing CRRT with IHD in patients who may transition from one to the other over time, a quasi-intention-to-treat approach may be rational. Our study addressed this issue in this way and provides a comprehensive adjusted analysis. In addition, in those patients who, in both trials, were treated with CRRT first because of a high cardiovascular SOFA score, subsequent care differed according to trial protocol only. This makes it possible to assess the adjusted association of a particular trial protocol with renal and patient outcomes, an analysis that has not been possible until now. A similar approach was taken in a recent study of patients involved in two trials of timing or RRT, where only patients allocated to early intervention were assessed [19]. Such study did not find an association between RRT modality and renal recovery. However, the sample size was almost a quarter that of our study, increasing the risk of type II error, and the analysis of recovery was limited by not taking duration of exposure and recovery among survivors into account.

### Clinical implications

Our findings imply that in the ATN and RENAL trials, after adjustment, receiving CRRT as the first modality of RRT was associated with faster recovery. They also imply that in those patients who went on to survive to at least 28 days, having received IHD as the first RRT modality was independently significantly associated with a greater probability of persistent RRT dependence, a finding which was robust to multiple sensitivity analyses. Finally, they imply that the treatment protocol had a strong association with renal and patient outcomes.

### Strengths and limitations

This is a large observational study and the first to use individual patient data from two high-quality large multicenter RCTs from the USA and ANZ. Thus, these findings have a degree of internal and external validity. Moreover, the data were collected independently for the purpose of the trials and without knowledge that this analysis would be performed. Thus, they carry no conceivable ascertainment bias. In addition, the methods used for the analysis considered the effect of death before the event of interest (end of RRT dependence) as a competing risk, adjusted analyses for multiple key baseline characteristics, and included several sensitivity analyses and re-assessments in different cohort, which repeatedly showed the same pattern of delayed and/or decreased recovery. Finally, this study is the first to analyze the association between treatment protocol and renal and patient outcomes in CRRT-first patients.

We acknowledge several limitations. First, our study is observational in nature. Thus, unmeasured confounding could have affected the results. We can only report associations, not causation, and the findings can only be considered hypothesis generating. Second, in the RENAL trial, all patients received only CRRT, while, in the ATN trial, they received both CRRT and IHD depending on their hemodynamic state. Thus, all IHD patients are from the ATN trial, creating additional bias. Such possible “trial protocol effect” was supported by the analysis of patients with high cardiovascular SOFA scores. Third, independent of trial design, time from ICU admission to randomization was longer in the ATN trial. However, illness severity scores and laboratory tests at RRT initiation were nearly identical and time-to-intervention difference was adjusted for. Fourth, although we mainly focused on RRT dependence at day 28 as the primary outcome in order to maximize power, we acknowledge that there is no consensus on the definition of persistent RRT dependence after AKI. Finally, the association between treatment protocol and renal and patient outcomes in CRRT-first patient may not reflect just the impact of the treatment protocol itself but also the impact of other unmeasured practice variation differences between the USA and ANZ.

## Conclusions

In critically ill patients with AKI treated with RRT, compared with the use of IHD as first treatment modality, after adjustment for confounders, use of CRRT as first treatment modality was not associated with decreased RRT dependence at day 28. However, among survivors to at least day 28, use of CRRT as first modality was associated with decrease risk of RRT dependence at day 28 and more RRT-free days at day 28. These findings were supported by multiple sensitivity analyses and appeared to reflect not only the choice of modality but, in CRRT-first patients, the possible impact of trial protocol. These findings provide additional detailed epidemiological information to inform clinical decision-making regarding choice of treatment modality and care protocols in critically ill patients with severe AKI.

## Supplementary Information

**Additional file 1. **Supplementary Methods, Supplementary Tables 1-13 and Supplementary Figures 1-8.

## Data Availability

The datasets used and/or analyzed during the current study are available from the corresponding author on reasonable request.

## Abbreviations

AKI: Acute kidney injury
RRT: Renal replacement therapy
CRRT: Continuous renal replacement therapy
IHD: Intermittent hemodialysis
RCT: Randomized controlled trial
RENAL: Randomized evaluation of normal versus augmented level of RRT
ATN: Acute renal failure trial network
ICU: Intensive care unit
ANZ: Australia and New Zealand
APACHE: Acute Physiology and Chronic Health Evaluation
SHR: Sub-distribution hazard ratios
CI: Confidence interval
OR: Common odds ratio
ORs: Odds ratios
HRs: Hazard ratios
CPBS: Covariate-balancing propensity score
SOFA Score: Sequential organ failure assessment score
USA: United States of America
