# Supplementary material for: Renal outcomes according to renal replacement therapy modality and treatment protocol in the ATN and RENAL trials
Source: Crit Care. 2022 Sep 6;26:269. doi: 10.1186/s13054-022-04151-5 (PMC9450407; doi:10.1186/s13054-022-04151-5)

Renal Replacement Therapy Dependence in Critically Ill Patients According to Continuous or Intermittent Renal Replacement Therapy

ONLINE SUPPLEMENT

eMETHODS

Sensitivity analysis

For all analysis below (considering the weighted regressions), the renal replacement therapy dependence at day 28 was assessed using a cause-specific hazard model, with the dependence at day 28 as the event of interest.

Inverse probability of treatment weighting

In this method, the treatment effect is estimated in a population whose distribution of risk factors is equal to that found in all study subjects. The calculation of the IPTW (*inverse probability of treatment weighted)*

was done according to Eq. 1.[1]

$$IPTW_T = \frac{1}{\hat{e}(X)} \; ; \; IPTW_{UN} = \frac{1}{1 - \hat{e}(X)} \quad (Eq. 1)$$

Where $IPTW_T$ is the IPTW for high CRRT group patients, $IPTW_{UN}$ is the IPTW for IHD group patients and ê(X) is the covariate balancing propensity score.

Stabilized inverse probability of treatment weighted

It may happen that treated subjects have a covariate balance propensity score near 0 or that untreated subjects have a covariate balancing propensity score near 1, making the relative IPTW excessively high and unstable. Computationally, as in any weighted regression, unstabilized IPTW changes the sample size of the original sample, generating an underestimate of the variance of the estimate of the effect, producing inappropriately narrow confidence intervals and leading to the lack of control of the probability of a type I error.[2]

Stabilized inverse probability of treatment weight (SIPTW) can be obtained by multiplying the IPTW by the marginal probability of receiving the actual treatment

received. Moreover, it preserves the sample size of the original data, produces appropriate estimation of the variance of the main effect, and adequately controls the type I error rate. The calculation of the IPTW was done according to Eq. 2.[2]

$$SIPTW_T = \frac{p}{\hat{e}(X)}\ ;\ SIPTW_{UN} = \frac{1-p}{1-\hat{e}(X)}\quad (Eq.2)$$

Where $SIPTW_T$ is the SIPTW for CRRT group patients, $SIPTW_{UN}$ is the SIPTW for IHD group patients, *p* is the probability of treatment without considering covariates (defined as n_t / N) and ê(X) is the covariate balancing propensity score.

References

1. Xu S, Ross C, Raebel MA, Shetterly S, Blanchette C, Smith D. Use of stabilized inverse propensity score as weight to directly estimate relative risk and its confidence intervals. Value Health. 2010;2:273–7.

2. Kurth T, Walker AM, Glynn RJ, et al. Results of Multivariable Logistic Regression, Propensity Matching, Propensity Adjustment, and Propensity-based Weighting under Conditions of Nonuniform Effect. Am J Epidemiol 2006; 163:262-70.

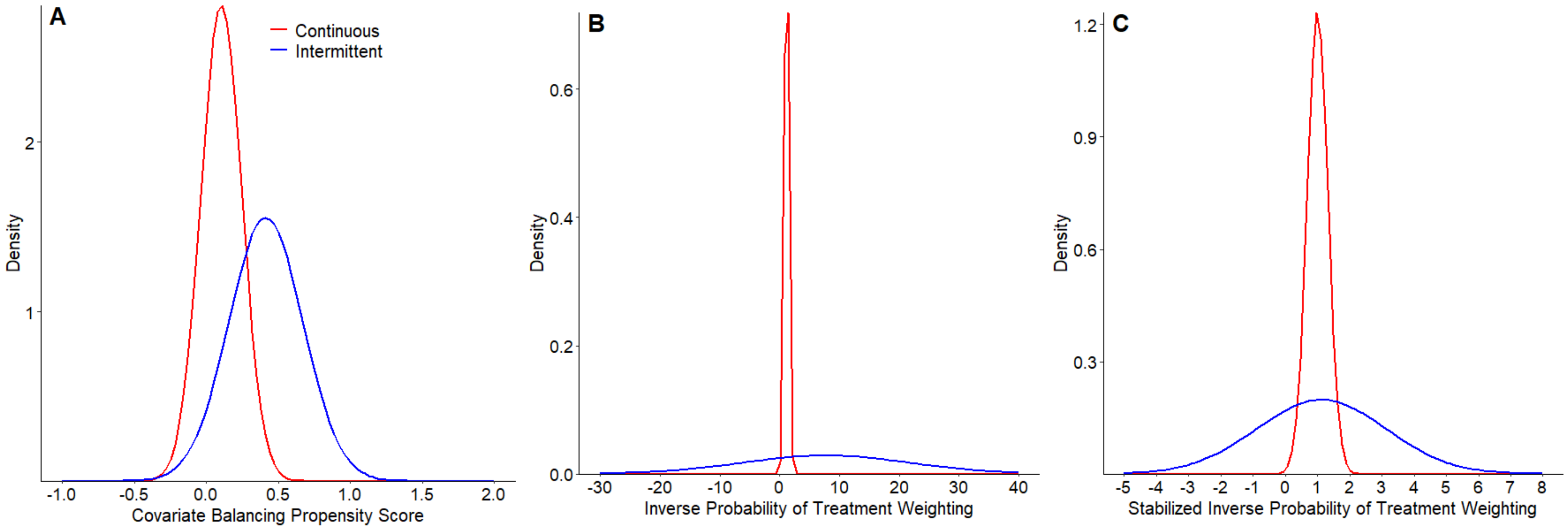

Probability density function of the: A) Covariate balancing propensity score, B) IPTW, and C) SIPTW.

eTable 1 - Rate of Missing Data According to the Groups

	Overall (*n* = 2542)	CRRT (*n* = 2175)	IHD (*n* = 367)
Age	0 (0)	0 (0)	0 (0)
Gender	0 (0)	0 (0)	0 (0)
Weight	0 (0)	0 (0)	0 (0)
Type of admission	0 (0)	0 (0)	0 (0)
APACHE III	2 (0.1)	2 (0.1)	0 (0)
Cardiovascular SOFA	3 (0.1)	3 (0.1)	0 (0)
Hours between randomization and start of treatment	0 (0)	0 (0)	0 (0)
Premorbid creatinine	831 (32.7)	747 (34.3)	84 (22.9)
Estimated glomerular filtration rate	831 (32.7)	747 (34.3)	84 (22.9)
Diabetes	1459 (57.4)	1459 (67.1)	0 (0)
Mechanical ventilation	0 (0)	0 (0)	0 (0)
Sepsis	1 (0)	1 (0)	0 (0)
Oliguria	0 (0)	0 (0)	0 (0)
Hyperkalemia	2 (0.1)	0 (0)	2 (0.5)
Acidemia	123 (4.8)	81 (3.7)	42 (11.4)
Urea > 25 mmol/L	2 (0.1)	0 (0)	2 (0.5)
Creatinine > 300 μmol/L	2 (0.1)	0 (0)	2 (0.5)
Urea	3 (0.1)	1 (0)	2 (0.5)
Creatinine	6 (0.2)	4 (0.2)	2 (0.5)
pH	179 (7)	137 (6.3)	42 (11.4)
Bicarbonate	14 (0.6)	12 (0.6)	2 (0.5)
Renal replacement dependence at day 28	0 (0)	0 (0)	0 (0)
Renal replacement dependence at day 60	34 (1.3)	26 (1.2)	8 (2.2)
ICU length of stay	141 (5.5)	129 (5.9)	12 (3.3)
Hospital length of stay	122 (4.8)	80 (3.7)	42 (11.4)
ICU mortality	0 (0)	0 (0)	0 (0)
Hospital mortality	0 (0)	0 (0)	0 (0)
28-day mortality	0 (0)	0 (0)	0 (0)
60-day mortality	28 (1.1)	24 (1.1)	4 (1.1)

eTable 2 - Baseline Characteristics of the Patients According to Renal Replacement Therapy Dependence at Day 28 in Survivors at the Latest Follow-Up Available

	Dependent (*n* = 433)	Independent (*n* = 1396)	*p* value
Age, years	62.0 (51.0 - 73.0)	65.2 (54.0 - 75.0)	0.001
Male gender - no. (%)	306 (70.7)	925 (66.3)	0.099
Weight, kilograms	82.6 (70.5 - 94.0)	80.0 (70.0 - 90.7)	0.111
Type of admission - no. (%)			< 0.001
Medical	204 (47.1)	925 (66.3)	
Surgical	199 (46.0)	428 (30.7)	
Other	30 (6.9)	43 (3.1)	
APACHE III	85.8 (70.4 - 103.0)	94.0 (77.0 - 111.0)	< 0.001
Cardiovascular SOFA	3.0 (0.0 - 4.0)	3.0 (1.0 - 4.0)	< 0.001
0	129 (29.8)	291 (20.9)	
1	62 (14.3)	169 (12.1)	
2	25 (5.8)	47 (3.4)	< 0.001
3	68 (15.7)	259 (18.6)	
4	149 (34.4)	628 (45.1)	
Hours between randomization and start of treatment	72.0 (24.0 - 144.0)	27.0 (8.0 - 72.0)	< 0.001
Premorbid creatinine, µmol/L	106.1 (79.6 - 140.0)	106.0 (79.6 - 138.0)	0.759
Estimated glomerular filtration rate, mL/min	60.8 (44.8 - 83.2)	60.6 (39.9 - 82.6)	0.410
Premorbid creatinine imputed, µmol/L*	100.7 (84.3 - 123.8)	100.9 (84.7 - 114.9)	0.961
Estimated glomerular filtration rate imputed, mL/min*	49.7 (37.1 - 61.1)	49.0 (41.2 - 59.9)	0.688
Diabetes - no. (%)	79 (27.7)	123 (31.9)	0.284
At baseline - no. (%)			
Mechanical ventilation	325 (75.1)	990 (70.9)	0.107
Sepsis	223 (51.5)	734 (52.6)	0.736
Oliguria	307 (70.9)	874 (62.6)	0.002
Hyperkalemia	21 (4.9)	83 (5.9)	0.463
Acidemia	46 (11.4)	379 (28.1)	< 0.001
Urea > 25 mmol/L	171 (39.6)	558 (40.0)	0.922
Creatinine > 300 µmol/L	240 (55.6)	734 (52.6)	0.310
High intensity group - no. (%)	232 (53.6)	665 (47.6)	0.035
Laboratory tests at baseline			
Urea, mmol/L	20.4 (13.9 - 30.2)	20.9 (14.1 - 30.6)	0.916
Creatinine, µmol/L	318.2 (229.8 - 433.2)	309.4 (220.0 - 419.2)	0.133
pH	7.34 (7.28 - 7.41)	7.30 (7.21 - 7.37)	< 0.001
Bicarbonate, mmol/L	21.0 (17.0 - 24.0)	19.0 (16.0 - 23.0)	< 0.001
Process of care			
Maximum length of follow-up	27.0 (15.0 - 28.0)	9.0 (5.0 - 15.0)	< 0.001
Days receiving CRRT	7.0 (3.0 - 14.0)	4.0 (2.0 - 6.0)	< 0.001
Days receiving IHD	6.0 (0.0 - 13.0)	0.0 (0.0 - 0.0)	< 0.001
Days until first IHD session	5.0 (1.0 - 10.0)	4.0 (1.0 - 7.0)	< 0.001
Modality in the first day after randomization			< 0.001
CRRT	298 (73.4)	1132 (88.6)	

eTable 2 - Baseline Characteristics of the Patients According to Renal Replacement Therapy Dependence at Day 28 in Survivors at the Latest Follow-Up Available

	**Dependent (*n* = 433)**	**Independent (*n* = 1396)**	***p* value**
IHD	108 (26.6)	145 (11.4)	
Modality in the second day after randomization			< 0.001
CRRT	314 (76.4)	1174 (90.8)	
IHD	97 (23.6)	119 (9.2)	
Modality in the third day after randomization			< 0.001
CRRT	308 (76.8)	1028 (89.8)	
IHD	93 (23.2)	117 (10.2)	
Exclusive modality**			< 0.001
CRRT	131 (58.7)	1026 (88.1)	
IHD	92 (41.3)	138 (11.9)	

Data are median (quartile 25% - quartile 75%) or No (%). Percentages may not total 100 because of rounding. Denominators are shown when the overall sample size was not available.

Abbreviations: CRRT: continuous renal replacement therapy; IHD: intermittent hemodialysis; APACHE, Acute Physiology and Chronic Health Evaluation; SOFA: Sequential Organ Failure Assessment; ICU: intensive care unit.

* Missing values in creatinine imputed according to age and gender (Tiao JYH, *et al*. Cardiovascular Surgery 2002;10:445-51).

** Defined as patients who received only one type of therapy during the available follow-up.

eTable 3 - Process of Care According to the Groups

	CRRT (*n* = 2175)	IHD (*n* = 367)	SMD	*p* value
Process of care				
Maximum length of detailed follow-up	9.0 (4.0 - 17.0)	12.0 (5.0 - 23.0)	0.025	0.005
Days receiving CRRT	5.0 (3.0 - 8.0)	0.0 (0.0 - 0.0)	1.195	< 0.001
Days receiving IHD	0.0 (0.0 - 0.0)	6.0 (2.0 - 11.5)	1.209	< 0.001
Days until first IHD session	7.0 (5.0 - 10.0)	1.0 (1.0 - 1.0)	1.772	< 0.001
Modality in the first day after randomization			9.373	< 0.001
CRRT	1985 (98.8)	3 (1.0)		
IHD	25 (1.2)	310 (99.0)		
Modality in the second day after randomization			5.326	< 0.001
CRRT	2029 (99.2)	16 (5.7)		
IHD	16 (0.8)	264 (94.3)		
Modality in the third day after randomization			4.031	< 0.001
CRRT	1769 (98.9)	25 (9.7)		
IHD	19 (1.1)	233 (90.3)		
Exclusive modality*			---	---
CRRT	1684 (77.4)	---		
IHD	---	281 (76.6)		

Data are median (quartile 25% - quartile 75%) or No (%). Percentages may not total 100 because of rounding. Denominators are shown when the overall sample size was not available.

Abbreviations: CRRT: continuous renal replacement therapy; IHD: intermittent hemodialysis; SMD: standardized mean difference.

* Defined as patients who received only one type of therapy during the available follow-up.

eTable 4 - Sensitivity Analyses for the Primary Outcome

	Effect Estimate (95% CI)	*p* value
**Weighted models***		
Inverse probability of treatment weighting	SHR, 0.96 (0.92 to 0.99)	0.049
Stabilized inverse probability of treatment weighting	SHR, 0.96 (0.92 to 0.99)	0.041

Abbreviations: SHR: subdistribution hazard ratio; HR: hazard ratio

* All models are cause-specific hazard models, with the dependence at day 28 as the event of interest.

eTable 5 - Baseline Characteristics of the Patients in the Two Sensitivity Cohorts

	Exclusive Modality in the First Three Days[a]			Exclusive Modality Over the Available Follow-up		
	**CRRT (*n* = 1617)**	**IHD (*n* = 203)**	***p* value**	**CRRT (*n* = 1684)**	**IHD (*n* = 281)**	***p* value**
Age, years	64.8 (54.0 - 75.0)	61.0 (49.0 - 71.0)	< 0.001	66.0 (55.0 - 75.8)	62.0 (53.0 - 71.0)	< 0.001
Male gender - no. (%)	1068 (66.0)	155 (76.4)	0.004	1096 (65.1)	215 (76.5)	< 0.001
Weight, kilograms	80.0 (70.0 - 91.0)	81.1 (71.2 - 95.3)	0.286	80.0 (70.0 - 90.0)	83.6 (71.0 - 97.0)	0.004
Type of admission - no. (%)			0.011			< 0.001
Medical	947 (58.6)	108 (53.2)		1094 (65.0)	152 (54.1)	
Surgical	591 (36.5)	75 (36.9)		538 (31.9)	107 (38.1)	
Other	79 (4.9)	20 (9.9)		52 (3.1)	22 (7.8)	
APACHE III	95.1 (79.7 - 111.0)	73.5 (61.2 - 85.8)	< 0.001	99.0 (83.0 - 116.0)	76.6 (64.3 - 88.9)	< 0.001
Cardiovascular SOFA	4.0 (2.0 - 4.0)	0.0 (0.0 - 1.0)	< 0.001	4.0 (2.0 - 4.0)	0.0 (0.0 - 1.0)	< 0.001
0	210 (13.0)	146 (71.9)		215 (12.8)	177 (63.0)	
1	171 (10.6)	37 (18.2)		175 (10.4)	46 (16.4)	
2	55 (3.4)	16 (7.9)	< 0.001	42 (2.5)	22 (7.8)	< 0.001
3	331 (20.5)	3 (1.5)		315 (18.7)	13 (4.6)	
4	849 (52.5)	1 (0.5)		935 (55.6)	23 (8.2)	
Hours between randomization and start of treatment	45.0 (15.0 - 96.0)	96.0 (48.0 - 168.0)	< 0.001	26.0 (8.0 - 72.0)	72.0 (48.0 - 144.0)	< 0.001
Premorbid creatinine, μmol/L	97.2 (79.6 - 132.6)	97.2 (79.6 - 132.6)	0.396	103.5 (79.6 - 139.8)	101.7 (79.6 - 132.6)	0.070
Estimated glomerular filtration rate, mL/min	61.6 (43.9 - 84.4)	67.8 (51.0 - 96.0)	0.002	59.3 (39.7 - 80.3)	65.3 (49.4 - 92.7)	< 0.001
Premorbid creatinine imputed, μmol/L*	100.7 (84.3 - 114.9)	100.7 (81.4 - 123.8)	0.982	100.9 (84.7 - 114.9)	100.9 (80.7 - 123.8)	0.585
Estimated glomerular filtration rate imputed, mL/min*	49.6 (41.8 - 60.8)	50.3 (40.1 - 64.2)	0.517	48.8 (41.7 - 59.6)	49.0 (39.5 - 64.5)	0.225
Diabetes - no. (%)	164 (26.6)	68 (33.5)	0.071	97 (27.1)	102 (36.3)	0.016
At baseline - no. (%)						
Mechanical ventilation	1298 (80.3)	107 (52.7)	< 0.001	1303 (77.4)	160 (56.9)	< 0.001
Sepsis	896 (55.4)	120 (59.1)	0.354	891 (52.9)	162 (57.7)	0.158
Oliguria	1106 (68.4)	135 (66.5)	0.641	1073 (63.7)	191 (68.0)	0.190
Hyperkalemia	78 (4.8)	2 (1.0)	0.020	112 (6.7)	3 (1.1)	< 0.001

eTable 5 - Baseline Characteristics of the Patients in the Two Sensitivity Cohorts

	Exclusive Modality in the First Three Days[a]			Exclusive Modality Over the Available Follow-up		
	**CRRT (*n* = 1617)**	**IHD (*n* = 203)**	***p* value**	**CRRT (*n* = 1684)**	**IHD (*n* = 281)**	***p* value**
Acidemia	357 (23.1)	9 (5.1)	< 0.001	500 (30.4)	11 (4.5)	< 0.001
Urea > 25 mmol/L	632 (39.1)	61 (30.2)	0.018	668 (39.7)	86 (30.8)	0.006
Creatinine > 300 μmol/L	823 (50.9)	110 (54.5)	0.379	822 (48.8)	159 (57.0)	0.014
High intensity group - no. (%)	798 (49.4)	105 (51.7)	0.573	837 (49.7)	134 (47.7)	0.575
Laboratory tests at baseline						
Urea, mmol/L	20.4 (13.9 - 30.6)	18.2 (12.9 - 26.7)	0.014	20.4 (13.6 - 30.7)	19.6 (13.2 - 27.1)	0.038
Creatinine, μmol/L	300.6 (218.0 - 412.0)	305.0 (247.5 - 397.8)	0.371	291.7 (209.0 - 400.0)	309.4 (247.5 - 397.8)	0.056
pH	7.3 (7.2 - 7.4)	7.4 (7.3 - 7.4)	< 0.001	7.3 (7.2 - 7.4)	7.4 (7.3 - 7.4)	< 0.001
Bicarbonate, mmol/L	20.0 (16.0 - 23.0)	22.0 (18.0 - 25.0)	< 0.001	19.0 (15.0 - 22.9)	22.0 (19.0 - 25.1)	< 0.001

Data are median (quartile 25% - quartile 75%) or No (%). Percentages may not total 100 because of rounding. Denominators are shown when the overall sample size was not available.

Abbreviations: CRRT: continuous renal replacement therapy; IHD: intermittent hemodialysis; APACHE, Acute Physiology and Chronic Health Evaluation; SOFA: Sequential Organ Failure Assessment; ICU: intensive care unit; SMD: standardized mean difference.

[a] Patients who died or do not have information of modality in the first three days were excluded from this analysis.

* Missing values in creatinine imputed according to age and gender (Tiao JYH, *et al*. Cardiovascular Surgery 2002;10:445-51).

eTable 6 - Primary and Secondary Outcomes in the Cohort of Patients According to the Exclusive Modality Received in the First Three Days[a]

	CRRT (*n* = 1617)	IHD (*n* = 203)	Unadjusted Analysis		Adjusted Analysis[b]	
			Effect Estimate (95% CI)	*p* value	Effect Estimate (95% CI)	*p* value
**Primary outcome**						
RRT dependence at day 28 - no. (%)	277 (17.1)	82 (40.4)	SHR, 0.69 (0.62 to 0.76)	< 0.001	SHR, 0.92 (0.79 to 1.08)	0.330
Death before the event	403 (24.9)	33 (16.3)				
**Secondary outcomes**						
RRT dependence at day 60 - no. (%)	128 (8.0)	36 (18.2)	SHR, 0.69 (0.57 to 0.83)	< 0.001	SHR, 0.92 (0.72 to 1.18)	0.510
Death before the event	452 (28.4)	46 (23.2)				
RRT-free days at day 28	0.0 (0.0 - 21.0)	0.0 (0.0 - 17.0)	COR, 1.44 (1.09 to 1.91)	0.011	COR, 1.66 (1.17 to 2.36)	0.004
Mean ± SD	9.1 ± 10.6	7.0 ± 9.6				
ICU length of stay, days	11.0 (6.0 - 22.0)	14.0 (7.0 - 25.0)	SHR, 0.66 (0.56 to 0.78)*	< 0.001	SHR, 0.78 (0.62 to 0.98)*	0.032
In survivors	12.0 (7.0 - 22.0)	13.0 (6.0 - 24.0)				
Hospital length of stay, days	22.0 (11.0 - 39.0)	27.5 (17.0 - 42.2)	SHR, 0.56 (0.47 to 0.68)*	< 0.001	SHR, 0.72 (0.57 to 0.91)*	0.007
In survivors	30.0 (18.0 - 48.5)	30.0 (18.0 - 43.0)				
ICU mortality - no. (%)	576 (35.6)	37 (18.2)	OR, 2.48 (1.73 to 3.64)	< 0.001	OR, 1.55 (0.98 to 2.49)	0.066
Hospital mortality - no. (%)	700 (43.3)	45 (22.2)	OR, 2.68 (1.91 to 3.82)	< 0.001	OR, 1.65 (1.08 to 2.55)	0.021
28-day mortality - no. (%)	589 (36.4)	40 (19.7)	HR, 2.04 (1.48 to 2.81)**	< 0.001	HR, 1.25 (0.86 to 1.82)	0.245
60-day mortality - no. (%)	695 (43.5)	59 (29.5)	HR, 1.66 (1.27 to 2.16)**	< 0.001	HR, 1.14 (0.83 to 1.57)	0.418
**Sensitivity analysis**						
Among survivors- no. (%)						
RRT dependence at day 28	201 (23.0)	66 (46.8)	OR, 0.33 (0.23 to 0.49)	< 0.001	OR, 0.42 (0.26 to 0.70)	< 0.001
RRT dependence at day 60	121 (13.9)	36 (25.9)	OR, 0.46 (0.30 to 0.71)	< 0.001	OR, 0.66 (0.37 to 1.18)	0.162

Data are median (quartile 25% - quartile 75%) or No (%). Percentages may not total 100 because of rounding. Denominators are shown when the overall sample size was not available.

Abbreviations: CRRT: continuous renal replacement therapy; IHD: intermittent hemodialysis; ICU: intensive care unit; RRT: renal replacement therapy; SHR: subdistribution hazard ratio; MD: mean difference; RRT: renal replacement therapy; HR: hazard ratio; OR: odds ratio; COR: common odds ratio.

[a] Patients who died or do not have information of modality in the first three days were excluded from this analysis.

[b] All models adjusted for age, gender, weight, type of admission (medical, surgical or other), APACHE III, cardiovascular SOFA, hours between randomization and therapy, use of mechanical ventilation, presence of oliguria, presence of hyperkalemia, presence of sepsis, last bicarbonate, urea and creatinine before randomization, premorbid estimated glomerular filtration rate and intensity of treatment (as allocated in the original trials).

* ICU and hospital length of stay censored at day 60.

** *p* value for Schoenfeld residual is 0.360 for 28-day mortality and 0.330 for 60-day mortality.

eTable 7 - Primary and Secondary Outcomes in the Cohort of Patients According to the Exclusive Modality Received in the Available Follow-up

	CRRT (*n* = 1684)	IHD (*n* = 281)	Unadjusted Analysis		Adjusted Analysis[a]	
			Effect Estimate (95% CI)	*p* value	Effect Estimate (95% CI)	*p* value
**Primary outcome**						
RRT dependence at day 28 - no. (%)	131 (7.8)	92 (32.7)	SHR, 0.49 (0.43 to 0.56)	< 0.001	SHR, 0.57 (0.47 to 0.71)	< 0.001
Death before the event	527 (31.3)	51 (18.1)				
**Secondary outcomes**						
RRT dependence at day 60 - no. (%)	54 (3.2)	42 (15.3)	SHR, 0.44 (0.35 to 0.56)	< 0.001	SHR, 0.61 (0.43 to 0.86)	0.005
Death before the event	551 (33.1)	63 (23.0)				
RRT-free days at day 28	0.0 (0.0 - 23.0)	0.0 (0.0 - 19.0)	COR, 1.32 (1.04 to 1.68)	0.024	COR, 1.54 (1.14 to 2.10)	0.005
Mean ± SD	10.2 ± 11.3	8.3 ± 10.6				
ICU length of stay, days	8.0 (4.0 - 15.0)	13.0 (7.0 - 23.0)	SHR, 0.65 (0.57 to 0.74)*	< 0.001	SHR, 0.74 (0.62 to 0.90)*	0.002
In survivors	8.0 (5.0 - 16.0)	13.0 (7.0 - 23.0)				
Hospital length of stay, days	16.0 (7.0 - 33.0)	24.0 (16.0 - 41.0)	SHR, 0.53 (0.45 to 0.62)*	< 0.001	SHR, 0.56 (0.45 to 0.69)*	< 0.001
In survivors	25.0 (15.0 - 45.0)	27.0 (17.0 - 42.0)				
ICU mortality - no. (%)	720 (42.8)	58 (20.6)	OR, 2.87 (2.13 to 3.92)	< 0.001	OR, 2.21 (1.50 to 3.27)	< 0.001
Hospital mortality - no. (%)	859 (51.0)	69 (24.6)	OR, 3.20 (2.41 to 4.29)	< 0.001	OR, 2.44 (1.71 to 3.54)	< 0.001
28-day mortality - no. (%)	746 (44.3)	67 (23.8)	HR, 2.15 (1.67 to 2.76)**	< 0.001	HR, 1.46 (1.09 to 1.94)	0.010
60-day mortality - no. (%)	826 (49.6)	87 (31.4)	HR, 1.86 (1.49 to 2.31)**	< 0.001	HR, 1.39 (1.07 to 1.81)	0.012
**Sensitivity analysis**						
Among survivors - no. (%)						
RRT dependence at day 28	82 (10.2)	76 (40.0)	OR, 0.17 (0.12 to 0.25)	< 0.001	OR, 0.19 (0.11 to 0.33)	< 0.001
RRT dependence at day 60	47 (5.8)	42 (22.5)	OR, 0.21 (0.14 to 0.34)	< 0.001	OR, 0.34 (0.18 to 0.66)	0.001

Data are median (quartile 25% - quartile 75%) or No (%). Percentages may not total 100 because of rounding. Denominators are shown when the overall sample size was not available.

Abbreviations: CRRT: continuous renal replacement therapy; IHD: intermittent hemodialysis; ICU: intensive care unit; RRT: renal replacement therapy; SHR: subdistribution hazard ratio; MD: mean difference; RRT: renal replacement therapy; HR: hazard ratio; OR: odds ratio; COR: common odds ratio.

[a] All models adjusted for age, gender, weight, type of admission (medical, surgical or other), APACHE III, cardiovascular SOFA, hours between randomization and therapy, use of mechanical ventilation, presence of oliguria, presence of hyperkalemia, presence of sepsis, last bicarbonate, urea and creatinine before randomization, premorbid estimated glomerular filtration rate and intensity of treatment (as allocated in the original trials).

* ICU and hospital length of stay censored at day 60.

** *p* value for Schoenfeld residual is 0.850 for 28-day mortality and 0.110 for 60-day mortality.

eTable 8 - Baseline Characteristics According to Modality of Dialysis in Patients Surviving Until the Latest Follow-Up

	CRRT (*n* = 1110)	IHD (*n* = 220)	*p* value
Age, years	63.8 (50.9 - 73.6)	60.0 (48.0 - 69.0)	0.002
Male gender - no. (%)	721 (65.0)	163 (74.1)	0.011
Weight, kilograms	80.8 (70.0 - 91.3)	84.3 (71.2 - 97.5)	0.076
Type of admission - no. (%)			< 0.001
Medical	691 (62.3)	108 (49.1)	
Surgical	376 (33.9)	94 (42.7)	
Other	43 (3.9)	18 (8.2)	
APACHE III	93.0 (77.0 - 109.0)	73.5 (63.5 - 85.8)	< 0.001
Cardiovascular SOFA	3.0 (1.0 - 4.0)	0.0 (0.0 - 1.0)	< 0.001
0	188 (16.9)	140 (63.6)	
1	130 (11.7)	34 (15.5)	
2	41 (3.7)	16 (7.3)	< 0.001
3	233 (21.0)	7 (3.2)	
4	518 (46.7)	23 (10.5)	
Hours between randomization and start of treatment	25.0 (7.2 - 70.8)	72.0 (24.0 - 168.0)	< 0.001
Premorbid creatinine, µmol/L	100.0 (79.6 - 138.0)	106.1 (70.7 - 132.6)	0.153
Estimated glomerular filtration rate, mL/min	61.6 (41.9 - 84.9)	68.6 (50.9 - 95.5)	< 0.001
Premorbid creatinine imputed, µmol/L*	100.7 (84.7 - 110.0)	100.7 (79.6 - 123.8)	0.699
Estimated glomerular filtration rate imputed, mL/min*	50.3 (42.0 - 60.1)	50.7 (40.1 - 65.5)	0.333
Diabetes - no. (%)	78 (25.8)	80 (36.4)	0.013
At baseline - no. (%)			
Mechanical ventilation	802 (72.3)	118 (53.6)	< 0.001
Sepsis	549 (49.5)	132 (60.0)	0.005
Oliguria	718 (64.7)	140 (63.6)	0.826
Hyperkalemia	76 (6.8)	3 (1.4)	0.003
Acidemia	287 (26.7)	10 (5.2)	< 0.001
Urea > 25 mmol/L	433 (39.0)	64 (29.4)	0.009
Creatinine > 300 µmol/L	611 (55.0)	114 (52.3)	0.502
High intensity group - no. (%)	553 (49.8)	102 (46.4)	0.388
Laboratory tests at baseline			
Urea, mmol/L	20.4 (14.0 - 30.2)	18.7 (12.6 - 26.4)	0.005
Creatinine, µmol/L	320.0 (226.0 - 443.5)	300.6 (229.8 - 397.8)	0.125
pH	7.30 (7.21 - 7.37)	7.36 (7.29 - 7.42)	< 0.001
Bicarbonate, mmol/L	19.0 (15.0 - 23.0)	22.0 (18.5 - 26.0)	< 0.001

Data are median (quartile 25% - quartile 75%) or No (%). Percentages may not total 100 because of rounding. Denominators are shown when the overall sample size was not available.

Abbreviations: CRRT: continuous renal replacement therapy; IHD: intermittent hemodialysis; APACHE, Acute Physiology and Chronic Health Evaluation; SOFA: Sequential Organ Failure Assessment; ICU: intensive care unit.

* Missing values in creatinine imputed according to age and gender (Tiao JYH, *et al*. Cardiovascular Surgery 2002;10:445-51).

eTable 9 - Baseline Characteristics According to Modality of Dialysis in Patients with Cardiovascular SOFA ≤ 2 at Baseline

	CRRT (*n* = 599)	IHD (*n* = 301)	*p* value
Age, years	63.4 (52.0 - 74.0)	62.0 (51.0 - 71.0)	0.077
Male gender - no. (%)	393 (65.6)	230 (76.4)	0.001
Weight, kilograms	80.0 (70.0 - 90.9)	82.6 (71.3 - 95.7)	0.054
Type of admission - no. (%)			< 0.001
Medical	403 (67.3)	159 (52.8)	
Surgical	158 (26.4)	112 (37.2)	
Other	38 (6.3)	30 (10.0)	
APACHE III	90.0 (74.0 - 104.2)	73.5 (64.3 - 88.9)	< 0.001
Cardiovascular SOFA	1.0 (0.0 - 1.0)	0.0 (0.0 - 1.0)	< 0.001
0	298 (49.7)	212 (70.4)	
1	234 (39.1)	61 (20.3)	< 0.001
2	67 (11.2)	28 (9.3)	
Hours between randomization and start of treatment	33.0 (5.0 - 96.0)	96.0 (48.0 - 168.0)	< 0.001
Premorbid creatinine, μmol/L	106.1 (79.6 - 151.0)	97.2 (70.7 - 132.6)	0.005
Estimated glomerular filtration rate, mL/min	58.0 (36.8 - 82.3)	65.8 (49.7 - 93.8)	< 0.001
Premorbid creatinine imputed, μmol/L*	100.9 (84.3 - 123.8)	100.7 (79.6 - 123.8)	0.114
Estimated glomerular filtration rate imputed, mL/min*	48.8 (37.1 - 60.4)	49.0 (40.1 - 64.3)	0.086
Diabetes - no. (%)	49 (25.1)	107 (35.5)	0.019
At baseline - no. (%)			
Mechanical ventilation	353 (58.9)	171 (56.8)	0.591
Sepsis	260 (43.4)	171 (56.8)	< 0.001
Oliguria	361 (60.3)	202 (67.1)	0.054
Hyperkalemia	53 (8.8)	3 (1.0)	< 0.001
Acidemia	107 (18.6)	11 (4.2)	< 0.001
Urea > 25 mmol/L	321 (53.6)	96 (32.0)	< 0.001
Creatinine > 300 μmol/L	374 (62.4)	168 (56.0)	0.074
High intensity group - no. (%)	312 (52.1)	145 (48.2)	0.300
Laboratory tests at baseline			
Urea, mmol/L	26.4 (16.6 - 35.2)	19.3 (13.5 - 27.5)	< 0.001
Creatinine, μmol/L	360.0 (247.0 - 495.0)	309.4 (238.7 - 415.5)	< 0.001
pH	7.32 (7.25 - 7.39)	7.36 (7.29 - 7.41)	< 0.001
Bicarbonate, mmol/L	19.0 (15.2 - 23.0)	22.0 (18.0 - 25.1)	< 0.001

Data are median (quartile 25% - quartile 75%) or No (%). Percentages may not total 100 because of rounding. Denominators are shown when the overall sample size was not available.

Abbreviations: CRRT: continuous renal replacement therapy; IHD: intermittent hemodialysis; APACHE, Acute Physiology and Chronic Health Evaluation; SOFA: Sequential Organ Failure Assessment; ICU: intensive care unit.

* Missing values in creatinine imputed according to age and gender (Tiao JYH, *et al*. Cardiovascular Surgery 2002;10:445-51).

eTable 10 - Primary and Secondary Outcomes in the Cohort of Patients with Cardiovascular SOFA ≤ 2 at Baseline

	CRRT (*n* = 599)	IHD (*n* = 301)	Unadjusted Analysis		Adjusted Analysis[a]	
			Effect Estimate (95% CI)	*p* value	Effect Estimate (95% CI)	*p* value
**Primary outcome**						
RRT dependence at day 28 - no. (%)	116 (19.4)	100 (33.2)	SHR, 0.93 (0.82 to 1.06)	0.300	SHR, 0.99 (0.86 to 1.16)	0.990
Death before the event	107 (17.9)	70 (23.3)				
**Secondary outcomes**						
RRT dependence at day 60 - no. (%)	60 (10.2)	46 (15.7)	SHR, 0.99 (0.80 to 1.23)	0.930	SHR, 1.07 (0.85 to 1.34)	0.560
Death before the event	122 (20.7)	84 (28.7)				
ICU length of stay, days	8.0 (4.0 - 17.0)	13.0 (7.0 - 25.0)	SHR, 1.12 (0.96 to 1.31)*	0.160	SHR, 1.01 (0.84 to 1.23)*	0.890
In survivors	8.0 (4.0 - 16.0)	13.0 (7.0 - 25.0)				
RRT-free days at day 28	4.0 (0.0 - 22.0)	0.0 (0.0 - 17.0)	COR, 1.72 (1.32 to 2.25)	< 0.001	COR, 1.55 (1.14 to 2.13)	0.005
Mean ± SD	10.6 ± 11.3	7.4 ± 10.2				
Hospital length of stay, days	21.0 (11.0 - 38.0)	26.0 (16.0 - 41.0)	SHR, 0.87 (0.72 to 1.04)*	0.130	SHR, 0.82 (0.66 to 1.01)*	0.670
In survivors	25.0 (15.0 - 42.0)	28.5 (17.2 - 43.8)				
ICU mortality - no. (%)	157 (26.2)	77 (25.6)	OR, 1.03 (0.75 to 1.42)	0.839	OR, 0.96 (0.66 to 1.41)	0.855
Hospital mortality - no. (%)	221 (36.9)	87 (28.9)	OR, 1.43 (1.07 to 1.94)	0.017	OR, 1.13 (0.79 to 1.62)	0.487
28-day mortality - no. (%)	173 (28.9)	83 (27.6)	HR, 1.05 (0.81 to 1.37)**	0.703	HR, 0.84 (0.63 to 1.13)**	0.245
60-day mortality - no. (%)	216 (36.5)	107 (36.0)	HR, 1.02 (0.81 to 1.28)**	0.892	HR, 0.85 (0.65 to 1.10)**	0.221
**Sensitivity analysis**						
Among survivors - no. (%)						
RRT dependence at day 28	86 (24.0)	81 (42.6)	OR, 0.42 (0.29 to 0.62)	< 0.001	OR, 0.53 (0.33 to 0.50)	0.008
RRT dependence at day 60	54 (15.1)	46 (24.6)	OR, 0.54 (0.35 to 0.85)	0.007	OR, 0.61 (0.35 to 1.07)	0.082

Data are median (quartile 25% - quartile 75%) or No (%). Percentages may not total 100 because of rounding. Denominators are shown when the overall sample size was not available.

Abbreviations: CRRT: continuous renal replacement therapy; IHD: intermittent hemodialysis; ICU: intensive care unit; RRT: renal replacement therapy; SHR: subdistribution hazard ratio; MD: mean difference; RRT: renal replacement therapy; HR: hazard ratio; OR: odds ratio.; COR: common odds ratio.

[a] All models adjusted for age, gender, weight, type of admission (medical, surgical or other), APACHE III, cardiovascular SOFA, hours between randomization and therapy, use of mechanical ventilation, presence of oliguria, presence of hyperkalemia, presence of sepsis, last bicarbonate, urea and creatinine before randomization, premorbid estimated glomerular filtration rate and intensity of treatment (as allocated in the original trials).

* ICU and hospital length of stay censored at day 60.

** *p* value for Schoenfeld residual is 0.670 for 28-day mortality and 0.840 for 60-day mortality.

eTable 11 - Baseline Characteristics of Patients Receiving CRRT and with Cardiovascular SOFA ≥ 3 at Baseline

	ATN (n = 521)	RENAL (n = 1052)	p value
Age, years	61.0 (51.0 - 72.0)	67.6 (57.7 - 76.3)	< 0.001
Male gender - no. (%)	357 (68.5)	678 (64.4)	0.122
Weight, kilograms	82.0 (70.0 - 95.5)	80.0 (70.0 - 90.0)	0.004
Type of admission - no. (%)			< 0.001
Medical	224 (43.0)	722 (68.6)	
Surgical	240 (46.1)	330 (31.4)	
Other	57 (10.9)	0 (0.0)	
APACHE III	92.0 (76.6 - 107.4)	105.0 (88.0 - 122.0)	< 0.001
Cardiovascular SOFA			
3	159 (30.5)	266 (25.3)	0.032
4	362 (69.5)	786 (74.7)	
Hours between randomization and start of treatment	72.0 (48.0 - 144.0)	21.0 (8.0 - 48.0)	< 0.001
Premorbid creatinine, µmol/L	88.4 (70.7 - 114.9)	106.0 (80.0 - 150.0)	< 0.001
Estimated glomerular filtration rate, mL/min	70.2 (53.1 - 96.9)	56.4 (36.1 - 75.7)	< 0.001
Premorbid creatinine imputed, µmol/L*	91.5 (79.6 - 106.1)	101.0 (86.1 - 110.2)	< 0.001
Estimated glomerular filtration rate imputed, mL/min*	54.1 (43.8 - 68.0)	48.2 (41.8 - 58.6)	< 0.001
Diabetes - no. (%)	137 (26.3)	N/A	---
At baseline - no. (%)			
Mechanical ventilation	480 (92.1)	878 (83.5)	< 0.001
Sepsis	347 (66.6)	564 (53.6)	< 0.001
Oliguria	436 (83.7)	660 (62.7)	< 0.001
Hyperkalemia	7 (1.3)	63 (6.0)	< 0.001
Acidemia	32 (6.9)	431 (41.0)	< 0.001
Urea > 25 mmol/L	167 (32.1)	353 (33.6)	0.590
Creatinine > 300 µmol/L	294 (56.4)	417 (39.6)	< 0.001
High intensity group - no. (%)	262 (50.3)	509 (48.4)	0.511
Laboratory tests at baseline			
Urea, mmol/L	19.3 (13.6 - 27.5)	18.4 (12.7 - 28.1)	0.268
Creatinine, µmol/L	318.2 (238.7 - 424.3)	261.0 (192.0 - 357.0)	< 0.001
pH	7.35 (7.28 - 7.41)	7.26 (7.16 - 7.34)	< 0.001
Bicarbonate, mmol/L	21.0 (18.0 - 24.0)	18.0 (14.7 - 22.0)	< 0.001

Data are median (quartile 25% - quartile 75%) or No (%). Percentages may not total 100 because of rounding. Denominators are shown when the overall sample size was not available.

Abbreviations: CRRT: continuous renal replacement therapy; IHD: intermittent hemodialysis; APACHE, Acute Physiology and Chronic Health Evaluation; SOFA: Sequential Organ Failure Assessment; ICU: intensive care unit.

* Missing values in creatinine imputed according to age and gender (Tiao JYH, *et al*. Cardiovascular Surgery 2002;10:445-51).

eTable 12 - Process of Care According to the Groups

	ATN<br>(*n* = 521)	RENAL<br>(*n* = 1052)	SMD	*p* value
Process of care				
Maximum length of follow-up	9.0 (4.0 - 20.0)	9.0 (5.0 - 16.0)	0.136	0.072
Days receiving CRRT	7.0 (4.0 - 12.0)	4.0 (3.0 - 7.0)	0.498	< 0.001
Use of IHD overall	234 (44.9)	69 (6.6)	0.976	< 0.001
Days on IHD among those who received it	5.0 (2.0 - 9.0)	2.0 (1.0 - 4.0)	0.764	< 0.001
Use of IHD in survivors	142 / 198 (72.2)	47 / 553 (8.5)	1.708	< 0.001
Days on IHD in survivors overall	4.0 (0.0 - 8.0)	0.0 (0.0 - 0.0)	1.279	< 0.001
Days on IHD among survivors who received it	7.0 (3.0 - 10.0)	2.0 (1.0 - 4.5)	1.009	< 0.001

Data are median (quartile 25% - quartile 75%) or No (%). Percentages may not total 100 because of rounding. Denominators are shown when the overall sample size was not available.

Abbreviations: CRRT: continuous renal replacement therapy; IHD: intermittent hemodialysis; SMD: standardized mean difference.

* Defined as patients who received only one type of therapy during the available follow-up.

eTable 13 - Primary and Secondary Outcomes in the Cohort of Patients Receiving CRRT and with Cardiovascular SOFA ≥ 3 at Baseline

	ATN (*n* = 521)	RENAL (*n* = 1052)	Unadjusted Analysis: Effect Estimate (95% CI)	Unadjusted Analysis: *p* value	Adjusted Analysis[a]: Effect Estimate (95% CI)	Adjusted Analysis[a]: *p* value
**Primary outcome**						
RRT dependence at day 28 - no. (%)	114 (21.9)	90 (8.6)	SHR, 1.04 (0.88 to 1.24)	0.620	SHR, 0.95 (0.76 to 1.18)	0.640
Death before the event	259 (49.7)	251 (23.9)				
**Secondary outcomes**						
RRT dependence at day 60 - no. (%)	55 (10.6)	30 (2.9)	SHR, 1.21 (0.88 to 1.66)	0.230	SHR, 1.12 (0.73 to 1.72)	0.600
Death before the event	291 (56.3)	259 (25.0)				
ICU length of stay, days	15.0 (8.0 - 28.0)	8.0 (3.2 - 14.0)	SHR, 0.59 (0.51 to 0.68)*	< 0.001	SHR, 0.54 (0.44 to 0.65)	< 0.001
In survivors	22.0 (12.8 - 33.0)	9.0 (6.0 - 17.0)				
RRT-free days at day 28	0.0 (0.0 - 0.0)	5.5 (0.0 - 23.0)	COR, 0.29 (0.23 to 0.36)	< 0.001	COR, 0.22 (0.16 to 0.29)	< 0.001
Mean ± SD	4.3 ± 8.3	10.7 ± 11.1				
Hospital length of stay, days	22.0 (11.0 - 37.0)	17.0 (6.0 - 36.0)	SHR, 0.72 (0.61 to 0.86)*	< 0.001	SHR, 0.72 (0.58 to 0.90)	0.004
In survivors	35.0 (22.0 - 49.0)	29.0 (16.0 - 52.0)				
ICU mortality - no. (%)	280 (53.7)	408 (38.8)	OR, 1.83 (1.48 to 2.27)	< 0.001	OR, 2.68 (2.02 to 3.59)	< 0.001
Hospital mortality - no. (%)	293 (56.2)	499 (47.4)	OR, 1.42 (1.15 to 1.76)	0.001	OR, 2.10 (1.58 to 2.80)	< 0.001
28-day mortality - no. (%)	280 (53.7)	420 (39.9)	HR, 1.51 (1.30 to 1.76)**	< 0.001	HR, 2.18 (1.80 to 2.64)	< 0.001
60-day mortality - no. (%)	322 (61.9)	465 (44.8)	HR, 1.59 (1.38 to 1.84)**	< 0.001	HR, 2.16 (1.81 to 2.59)	< 0.001
**Sensitivity analysis**						
Among survivors - no. (%)						
RRT dependence at day 28	83 (41.9)	63 (11.4)	OR, 5.61 (3.83 to 8.28)	< 0.001	OR, 6.87 (4.18 to 11.43)	< 0.001
RRT dependence at day 60	55 (28.2)	27 (4.9)	OR, 7.65 (4.70 to 12.74)	< 0.001	OR, 9.13 (4.91 to 17.50)	< 0.001

Data are median (quartile 25% - quartile 75%) or No (%). Percentages may not total 100 because of rounding. Denominators are shown when the overall sample size was not available.

Abbreviations: CRRT: continuous renal replacement therapy; IHD: intermittent hemodialysis; ICU: intensive care unit; RRT: renal replacement therapy; SHR: subdistribution hazard ratio; MD: mean difference; RRT: renal replacement therapy; HR: hazard ratio; OR: odds ratio.; COR: common odds ratio.

[a] All models adjusted for age, gender, weight, type of admission (medical, surgical or other), APACHE III, cardiovascular SOFA, hours between randomization and therapy, use of mechanical ventilation, presence of oliguria, presence of hyperkalemia, presence of sepsis, last bicarbonate, urea and creatinine before randomization, premorbid estimated glomerular filtration rate and intensity of treatment (as allocated in the original trials).

* ICU and hospital length of stay censored at day 60. ** *p* value for Schoenfeld residual is 0.840 for 28-day mortality and 0.370 for 60-day mortality.

eFigure 1 - Flowchart of the Included Patients

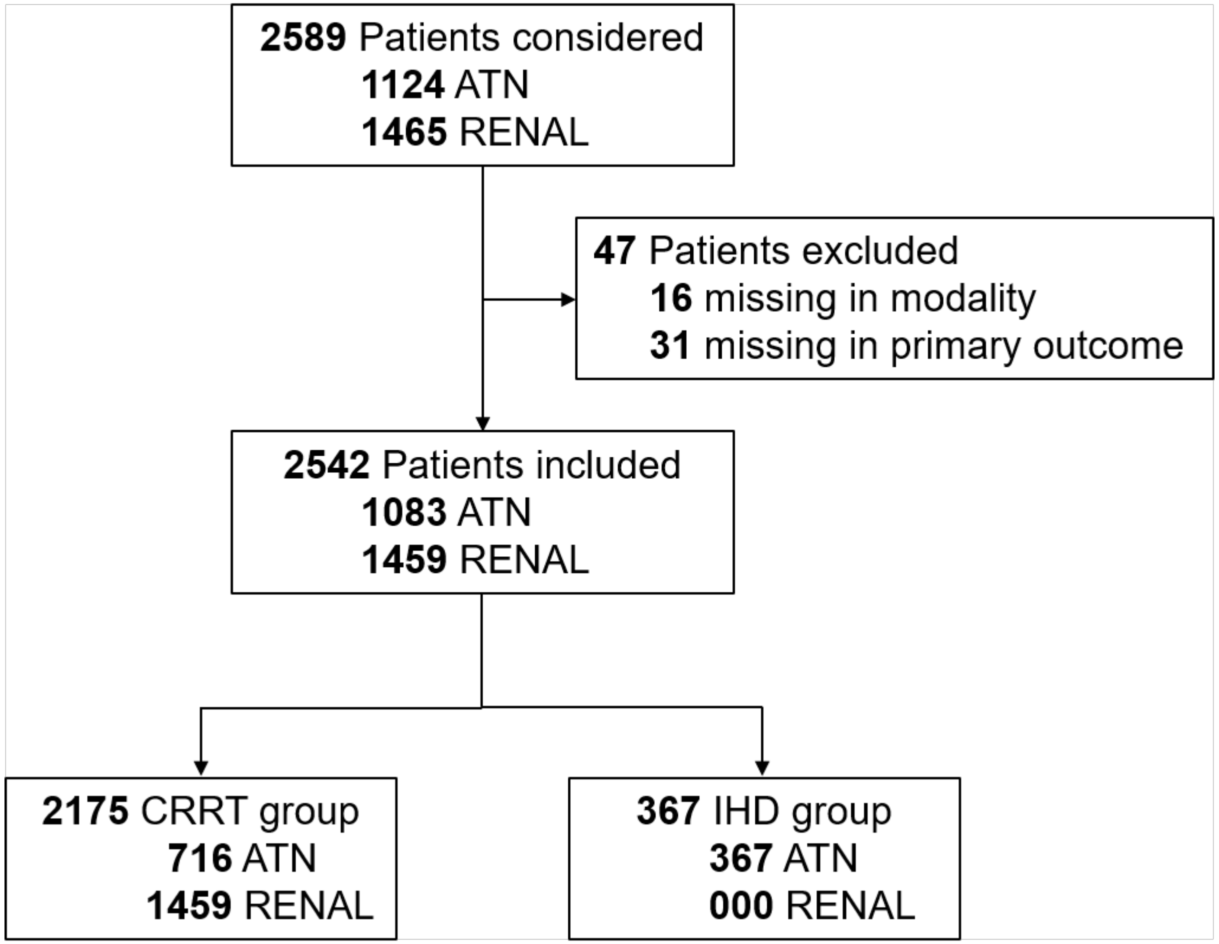

CRRT denotes continuous renal replacement therapy and IHD intermittent hemodialysis.

eFigure 2 - Percentage of Patients Receiving Continuous Renal Replacement Therapy Over the First 28 Days

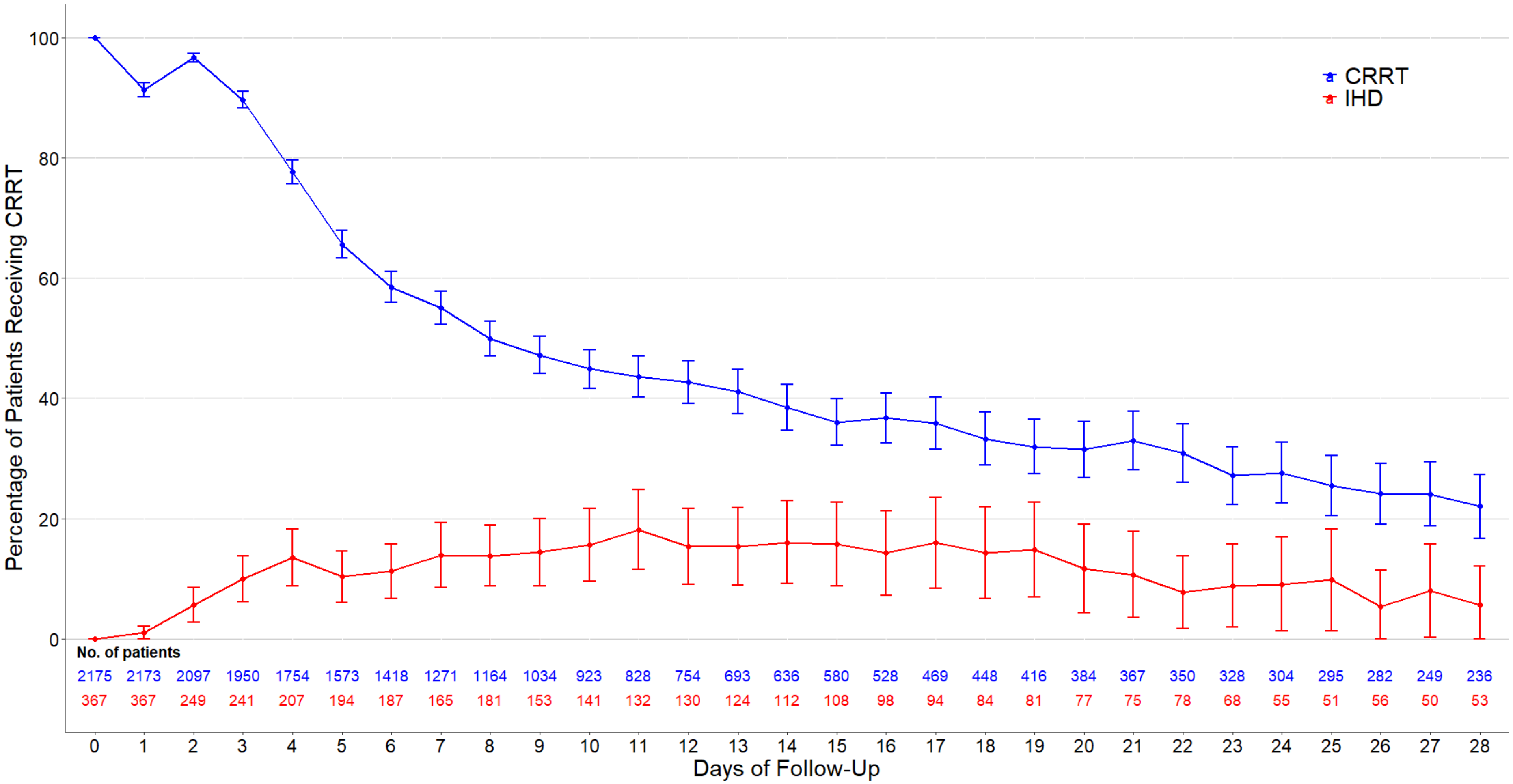

CRRT denotes continuous renal replacement therapy and IHD intermittent hemodialysis.

eFigure 3 - Percentage of Patients Receiving Intermittent Hemodialysis Over the First 28 Days

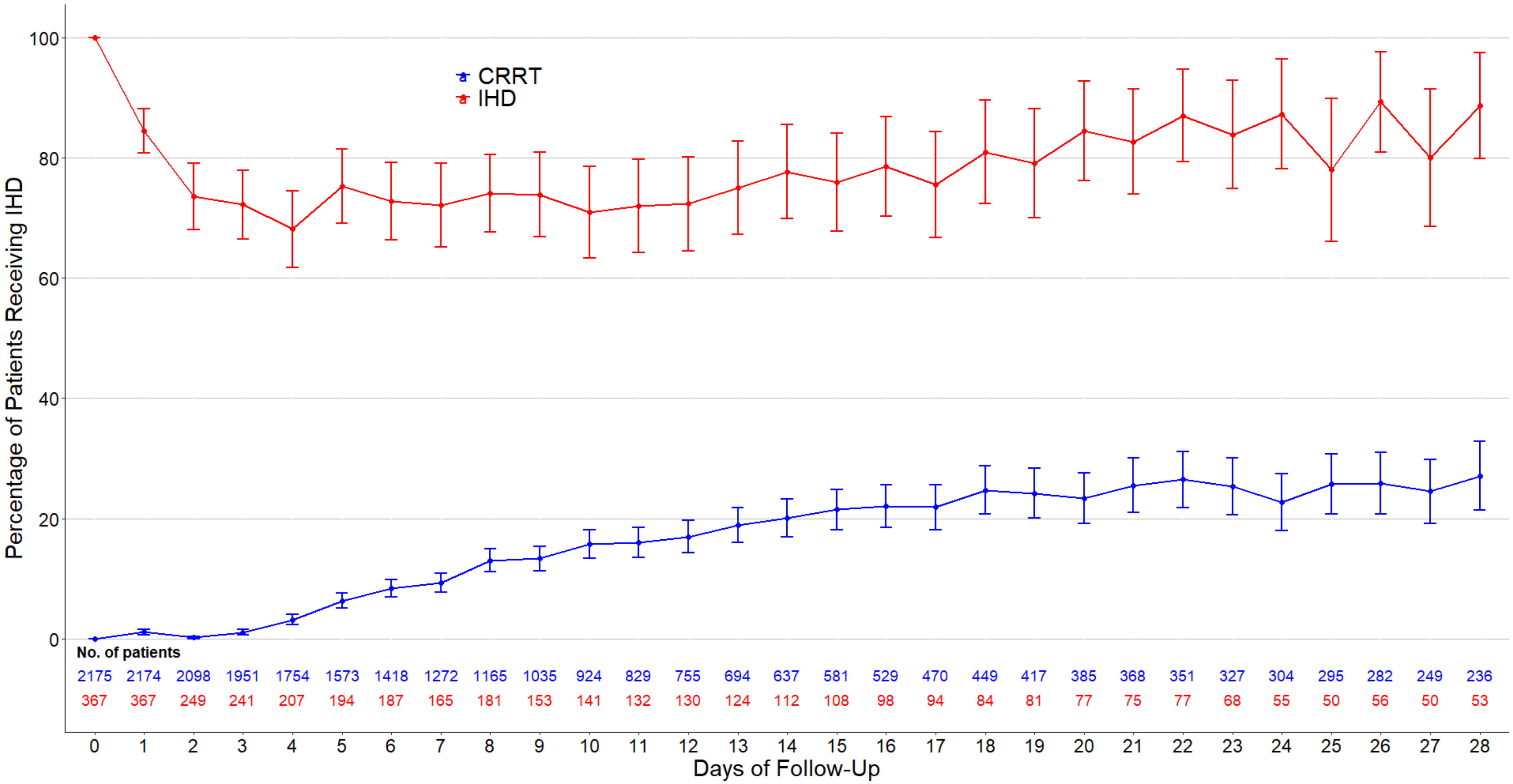

CRRT denotes continuous renal replacement therapy and IHD intermittent hemodialysis.

eFigure 4 - Percentage of Patients Receiving Continuous Renal Replacement Therapy or Intermittent Hemodialysis Over the First 28 Days among Patients Classified According to the Exclusive Modality Received in the First Three Days

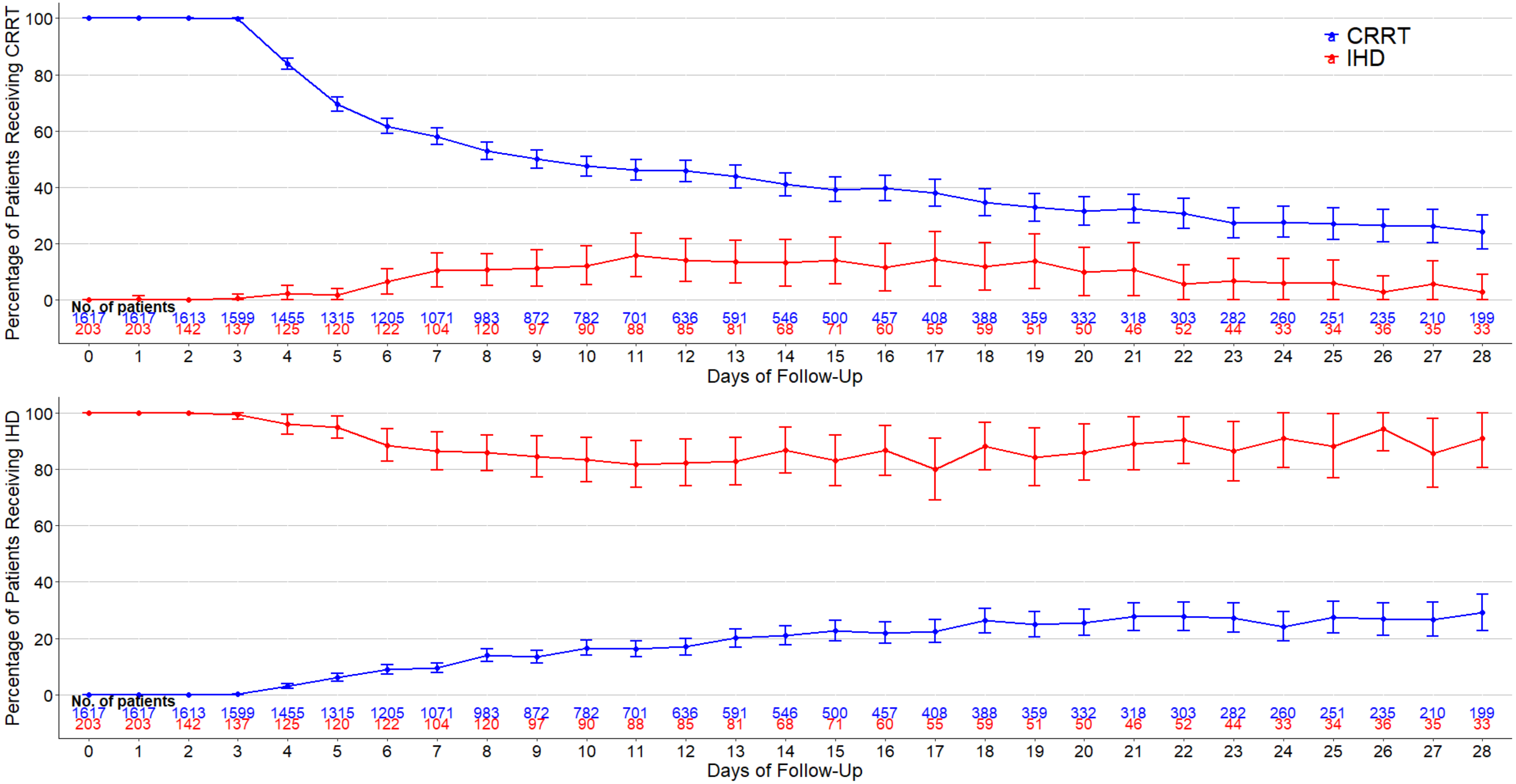

CRRT denotes continuous renal replacement therapy and IHD intermittent hemodialysis.

eFigure 5 - Percentage of Patients Receiving Continuous Renal Replacement Therapy or Intermittent Hemodialysis Over the First 28 Days among Patients Classified According to the Exclusive Modality Received in the Available Follow-Up

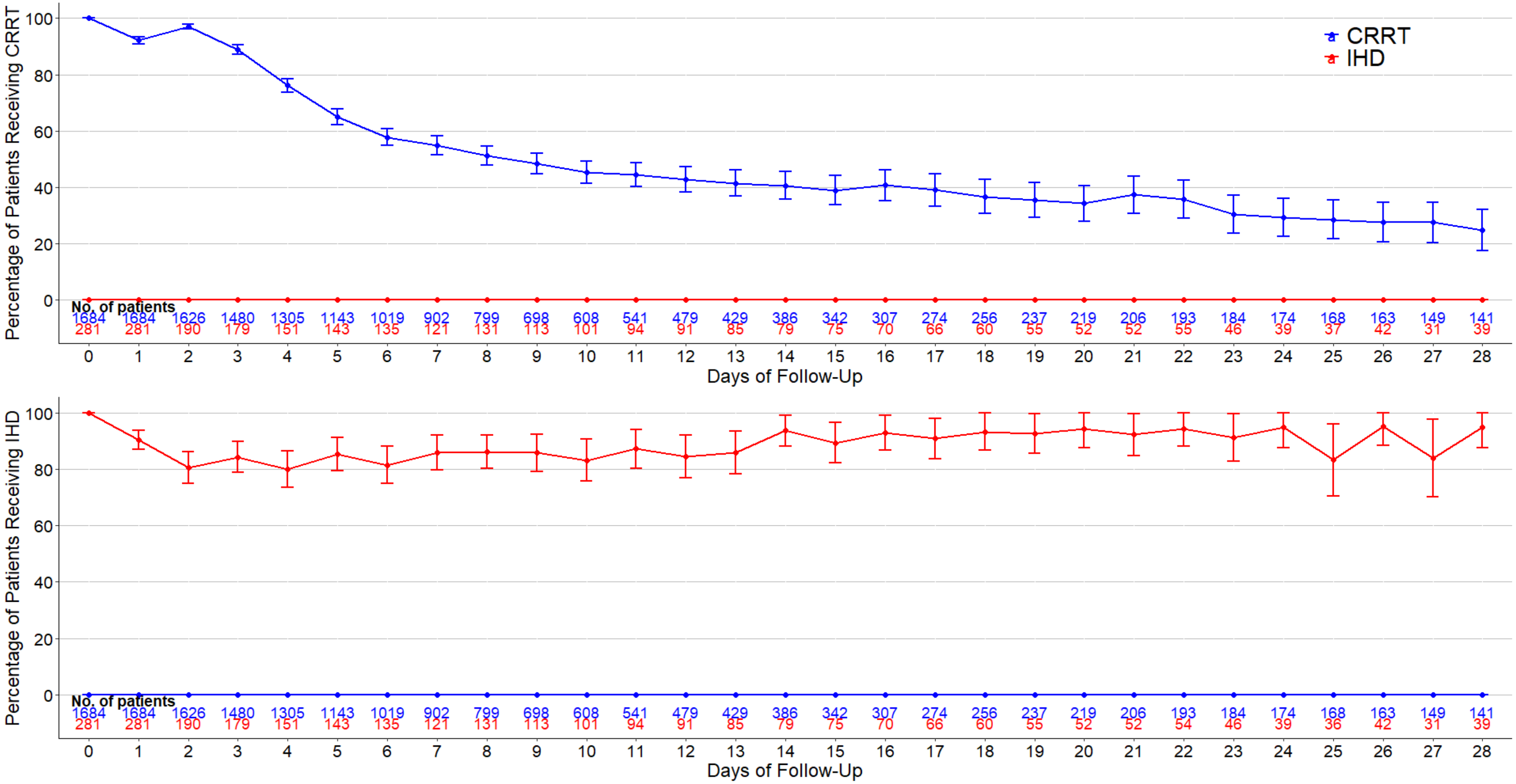

CRRT denotes continuous renal replacement therapy and IHD intermittent hemodialysis.

eFigure 6. The relationship between IHD/CRRT time ratio and RRT dependence at day 28 after randomization

A) IHD/CRRT time ratio at 2 weeks

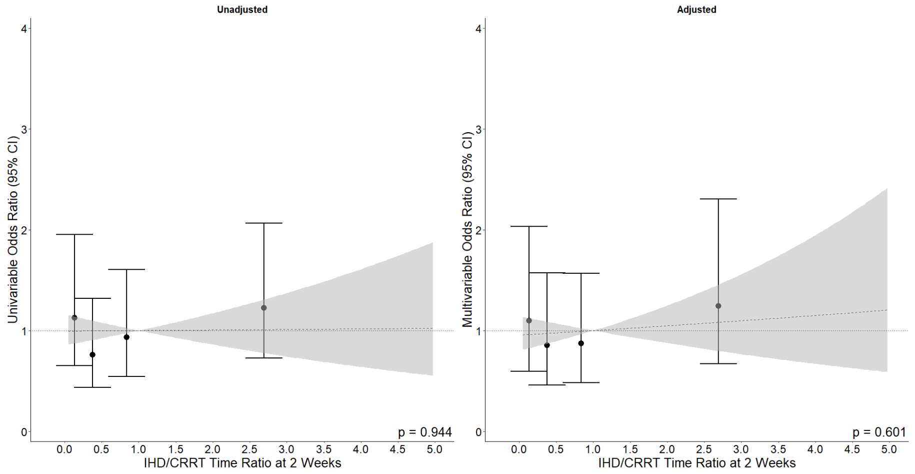

B) IHD/CRRT time ratio at 3 weeks

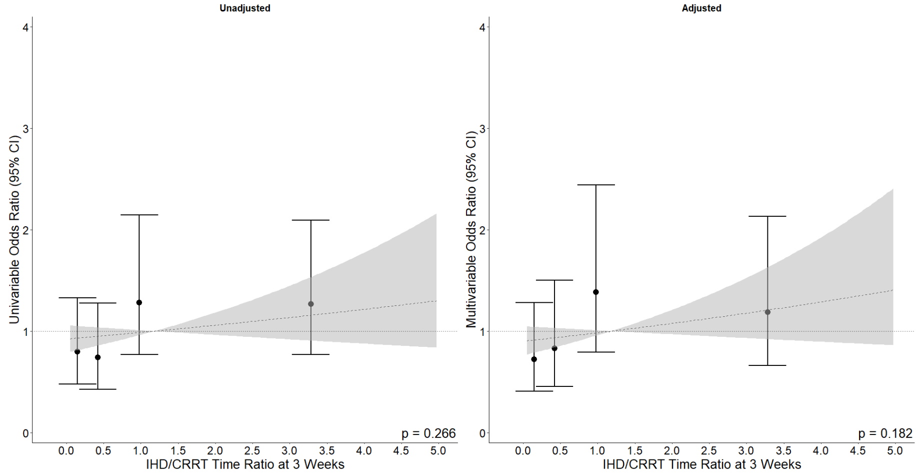

C) IHD/CRRT time ratio at 4 weeks

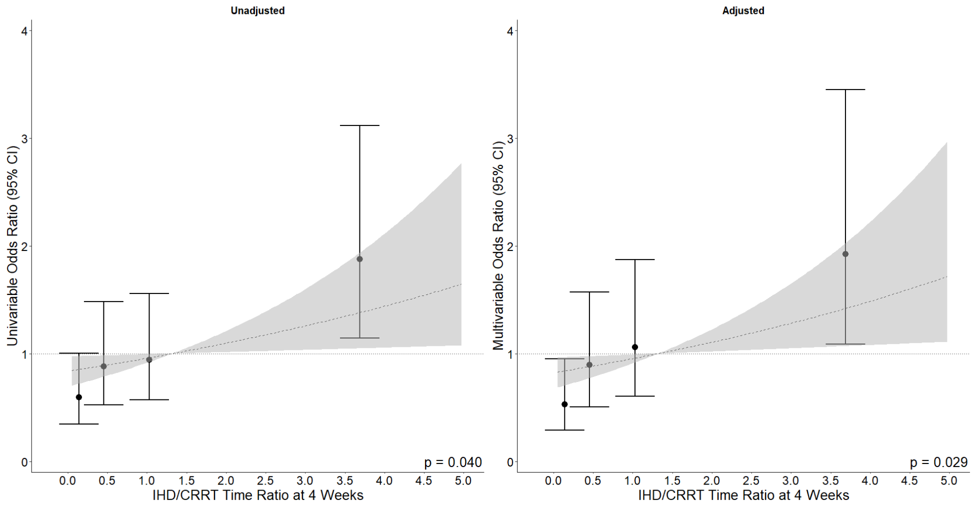

eFigure 7: Use of IHD Among All Patients with CVS SOFA≥3 at randomization

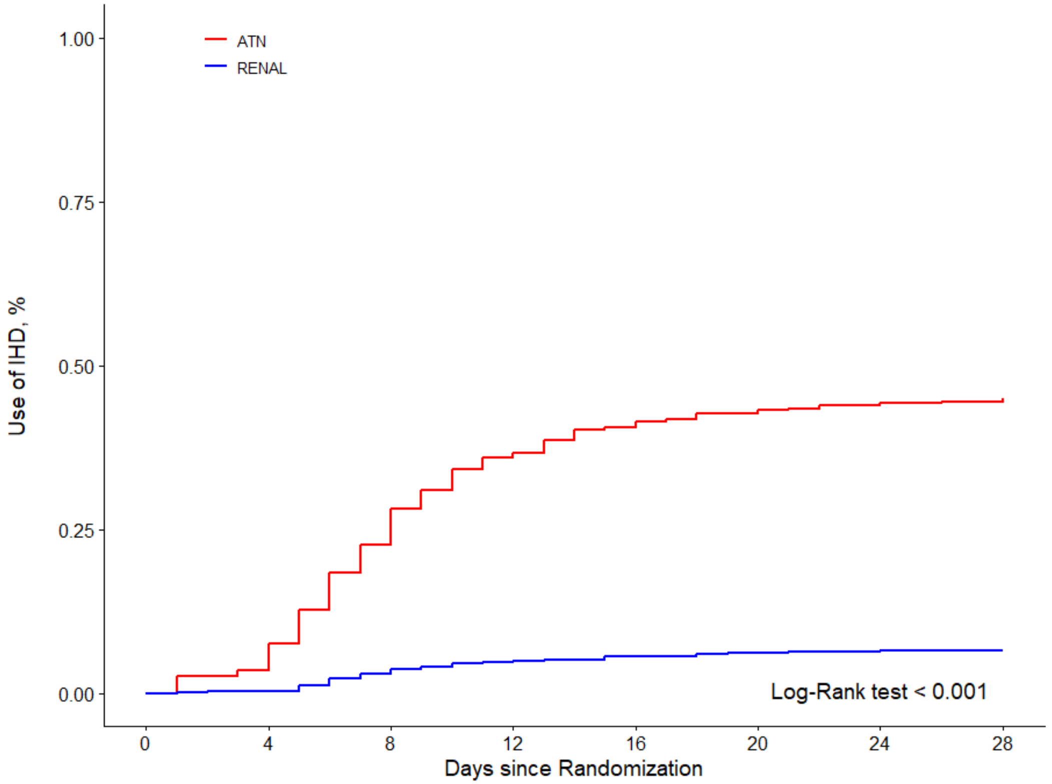

No. at Risk

ATN	521	503	403	334	310	299	292	289
RENAL	1052	1049	1021	1003	993	988	985	983

eFigure 8: Use of IHD Among Survivors with CVS SOFA≥3 at randomization

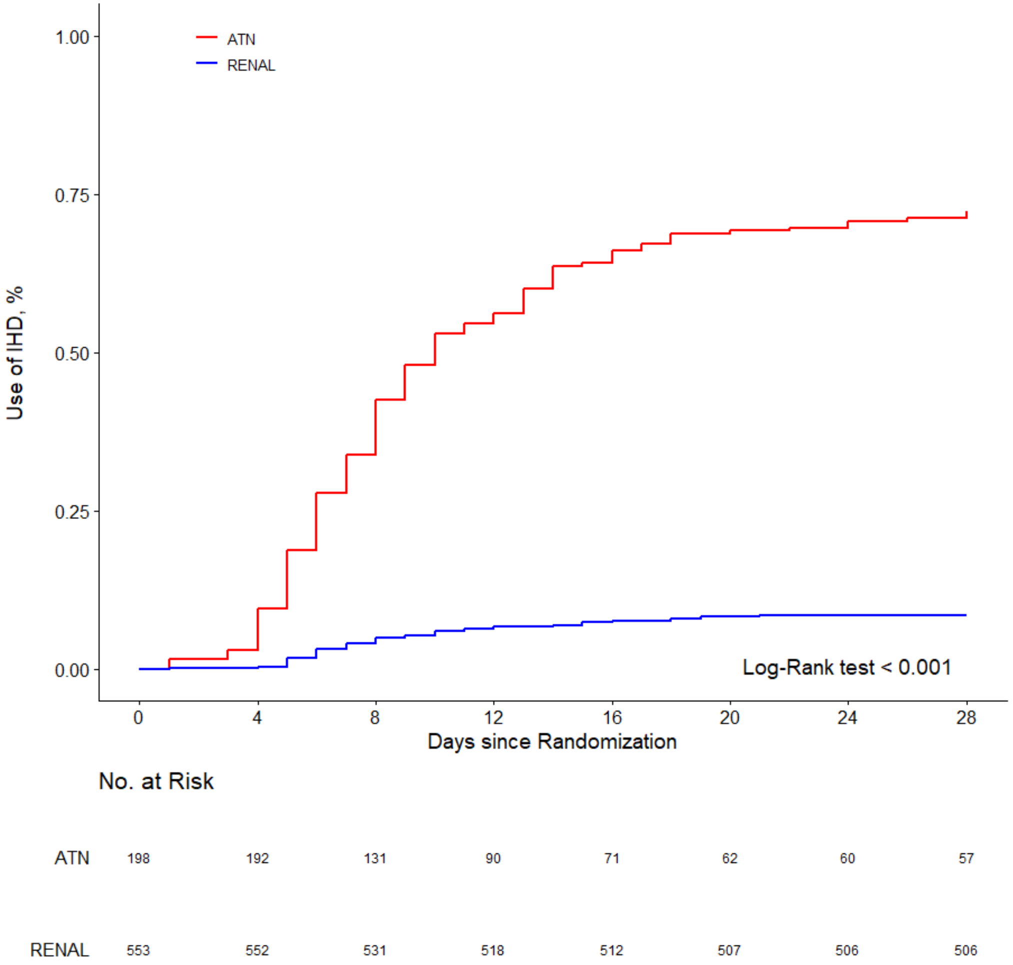

Supplement: Supplementary file 1 — Additional file 1. Supplementary Methods, Supplementary Tables 1-13 and Supplementary Figures 1-8. [file 13054_2022_4151_MOESM1_ESM.pdf]
